# Cytotoxic Necrotizing Factor 1 Downregulates CD36 Transcription in Macrophages to Induce Inflammation During Acute Urinary Tract Infections

**DOI:** 10.3389/fimmu.2018.01987

**Published:** 2018-08-31

**Authors:** Huan Yang, Qianqian Li, Changying Wang, Jingyu Wang, Junqiang Lv, Lei Wang, Zhi-Song Zhang, Zhi Yao, Quan Wang

**Affiliations:** ^1^Key Laboratory of Immune Microenvironment and Disease of the Educational Ministry of China, Tianjin Key Laboratory of Cellular and Molecular Immunology, Department of Immunology, School of Basic Medical Sciences, Tianjin Medical University, Tianjin, China; ^2^State Key Laboratory of Medicinal Chemical Biology, Tianjin Key Laboratory of Molecular Drug Research, Collaborative Innovation Center for Biotherapy, College of Pharmacy, Nankai University, Tianjin, China; ^3^Collaborative Innovation Center of Tianjin for Medical Epigenetics, Tianjin Medical University, Tianjin, China

**Keywords:** cytotoxic necrotizing factor 1, uropathogenic *Escherichia coli*, CD36, macrophages, inflammation

## Abstract

Urinary tract infections (UTIs) caused by uropathogenic *Escherichia coli* (UPEC) induce cystitis, pyelonephritis, and can cause kidney scarring and failure if inflammation is not under control. The detailed effects of cytotoxic necrotizing factor 1 (CNF1), the key UPEC toxin, on the pathogenicity of UPEC remain unclear. CD36 is an important scavenger receptor, responsible for pathogen and apoptotic cell clearance, and plays an essential role in host immune defense and homeostasis. Regulation of CD36 by bacterial toxins has not been reported. In this study, using a pyelonephritis mouse model, CNF1 was observed to contribute to increasing neutrophils and bacterial titers in infected bladder and kidney tissues, resulting in severe inflammation and tissue damage. CD36 expression in macrophages was found to be decreased by CNF1 *in vitro* and *in vivo*. We demonstrated that CNF1 attenuated CD36 transcription by decreasing expressions of its upstream transcription factors LXRβ and C/EBPα and their recruitment to the CD36 promotor. In addition, Cdc42 was found to be involved in CNF1-mediated downregulation of LXRβ. Our study investigated the pathogenesis of *cnf1*-carrying UPEC, which affected host innate immune defenses and homeostasis through regulation of CD36 in macrophages during acute UTIs.

## Introduction

Urinary tract infections (UTIs) are one of the commonest bacterial infections, which affect more than 100 million people annually worldwide, and uropathogenic *Escherichia coli* (UPEC) is the leading cause of UTIs (1–4). UTIs caused by UPEC usually induce cystitis, and can develop to acute pyelonephritis, which may result in kidney scarring and failure, especially in childhood (5, 6).

The innate immune response plays an important role in host defenses during UTIs mediated by UPEC (7). Phagocytic cells (neutrophils and macrophages) are recruited, and a large panel of cytokines and chemokines are upregulated during the infection to contribute to the innate clearance of UPEC (8–10). Although inflammation triggered during innate immunity is necessary for bacterial clearance, it should be resolved to prevent severe tissue damage (11). For example, neutrophils and debris from the inflamed sites should be removed to enable the tissue to return to homeostasis (11). Unresolved inflammation during UTIs would cause chronic infection and irreversible renal damage (5, 6, 12).

Professional phagocytes, such as macrophages, deal with various kinds of particles, ranging from pathogens to apoptotic cells, via a diverse set of receptors. The prototypic receptors include Fcγ receptor recognizing IgG-opsonized pathogens, integrins contributing to CR3-mediated phagocytosis, and scavenger receptors recognizing and removing apoptotic cells and pathogens (13).

CD36 is one of the important scavenger receptors, present on many types of cells, especially on macrophages and endothelium, and mediates lots of biological processes including angiogenesis, atherosclerosis, and innate immunity (14). CD36 functions as a pattern recognition receptor on macrophages, mediating phagocytosis and elimination of foreign agents, such as bacterial and fungal pathogens (14–18). CD36 also recognizes endogenous ligands, such as apoptotic cells including neutrophils (19–21).

Cytotoxic necrotizing factor 1 (CNF1) is one of the key UPEC toxins. One-third of pyelonephritis isolates contain the *cnf1* gene, implying its role in kidney infection (12). CNF1 induces cell motility and efficient cell invasion by UPEC by activating Rho GTPases (22–26). Many reports have shown that CNF1-positive strains cause more inflammation in bladder, kidney, and prostate *in vivo* (27–31); however, some studies reported that CNF1 had no effect on inflammation (32). Thus, the detailed role and mechanism of CNF1 in host inflammation and bacterial burden during cystitis and pyelonephritis remain unclear.

In this study, using a pyelonephritis mouse model, we found CNF1 increased neutrophils and decreased UPEC clearance in infected bladder and kidney tissues, and downregulated CD36 transcription in macrophages. The mechanism by which CNF1 reduces CD36 transcription was investigated.

## Materials and methods

### Cell lines and reagents

The cell lines and their sources are as follows: 293T (ATCC CRL-3216), THP-1 (ATCC TIB-202), and RAW264.7 (ATCC SC-6003). The Rac1 inhibitor, EHT1864 (HY-16659), was purchased from MedChem Express (Monmouth Junction, NJ, USA). The Cdc42 inhibitor CID44216842, RhoA inhibitor CCG-1423, the proteasome inhibitors MG132 (M7449), Salvianolic acid B (SAB, 49724), and the latex beads (L2778) were purchased from Sigma-Aldrich (St. Louis, MO, USA). The proteasome inhibitor bafilomycin A1 (ab120497) was purchased from Abcam (Cambridge, UK). F-actin probes with Rhodamine Phalloidin (PHDR1) were purchased from Cytoskeleton (Denver, CO, USA). LDH was detected using a CytoTox-96 Non-Radioactive Cytotoxicity Assay Kit (G1780), purchased from Promega (Madison, WI, USA).

### Bacterial strains and growth conditions

Bacterial strains and plasmids used are listed in Table S1. *E. coli* strains were cultured at 37°C in Luria-Bertani (LB) medium under static conditions for 12 h with appropriate antibiotics when required, at the following concentrations: kanamycin at 50 μg/ml, ampicillin at 100 μg/ml. The *cnf1* gene from UPEC strain 11 was amplified by PCR and cloned into pET-28a (+) or pTRC99A, as reported previously (26). The Δ*cnf1* strain was generated by the substitution of *cnf1* with a *cat* gene using the lambda red recombination system (33, 34).

### Mouse peritoneal macrophage and BMDM preparation

Six- to-eight week-old female C57BL/6J mice purchased from the Academy of Military Medical Science (Beijing, China) were injected intraperitoneally with 2 ml of aged thioglycolate broth (Sigma-Aldrich). At 2 days post injection, the mice were euthanized. Peritoneal lavage was performed with 20 ml of ice-cold PBS and centrifuged at 200 × g for 5 min. Cells were resuspended in DMEM containing 10% FBS, 100 U/ml penicillin, and 0.1 mg/ml streptomycin for 1 h at 37°C to allow adhesion.

Bone marrow derived macrophages (BMDMs) were isolated from C57BL/6J mice, and cultured in Dulbecco's modified Eagle's medium/F12 with 10% heat inactivated fetal calf serum, penicillin (100 U/ml), streptomycin (100 μg/ml), and 10 ng/ml of murine M-CSF (Peprotech, St. Louis, MO, USA). Cells were allowed to grow for 6 days before use.

### Antibodies and western blotting

Antibodies were obtained from the following companies: anti-CNF1 (sc-52655, Santa Cruz Biotechnology, Santa Cruz, CA, USA), anti-LXRβ (ab28479, Abcam), anti-C/EBPα (8178, Cell Signaling Technology, Danvers, MA, USA), anti-HIF1α (20960-1-AP, Proteintech, Chicago, IL, USA), and anti-CD36 (18836-1-AP, Proteintech). Whole cell lysates were prepared using RIPA lysis buffer (Millipore, Billerica, MA, USA), adding complete protease inhibitors (Roche, Basel, Switzerland). The protein concentration was determined using the BCA Protein Assay Kit (ThermoFisher, Waltham, MA, USA). Approximately 30 μg of cell lysates were used for loading. Antibody binding was revealed using an HRPA conjugated anti-rabbit IgG (Sigma-Aldrich) or anti-mouse IgG (Sigma-Aldrich). Antibody complexes were detected using Immobilon Western Chemiluminescent HRP Substrate (Millipore) and exposure to Tanon-5200 machine.

### Rho GTPase activation assays

RAW264.7 cells were seeded in 10 cm dishes. After corresponding treatment, Cdc42, Rac1, and RhoA activation was measured using the respective Activation Assay Biochem Kits (Cdc42: BK034; Rac1: BK035; RhoA: BK036, Cytoskeleton) according to the manufacturer's protocol.

### Immunofluorescence analysis of cells

Cells were grown on a Lab-Tek chambered coverglass, fixed with 4% paraformaldehyde for 30 min, permeabilized with 0.5% Triton X-100 for 10 min and blocked with PBS containing 10% goat serum for 1 h at room temperature. Samples were incubated with primary antibody (*E. coli* LPS antibody, Abcam, ab35654, 1:200; C/EBPα antibody, Cell Signaling Technology, 8178, 1:200; LXRβ antibody, Abcam, ab28479, 1:200; CD36 antibody; Proteintech, 18836-1-AP, 1:200) in PBS containing 10% goat serum at 4°C overnight, washed five times with PBS, incubated with Alexa Fluor 488/594-labeled second antibody (Proteintech) at room temperature for 1 h. Then 100 nM rhodamine phalloidin was added and incubated at room temperature for 30 min, and DAPI was added for 10 min. Cells were imaged using a confocal fluorescence microscope (FV1000-D, Olympus, Tokyo, Japan).

### ChIP-qPCR

The Chromatin Immunoprecipitation (ChIP) assay was performed using a SimpleChIP^®^ Plus Sonication Chromatin IP Kit (Cell Signaling Technology) according to the manufacturer's protocol. Cells were cross-linked for 20 min with 1% formaldehyde. Cells were pelleted and resuspended in lysis buffer and sonicated for 30 cycles. Then immunoprecipitation was performed using the anti-LXRβ antibody (Abcam, ab28479, 5 μg), anti-C/EBPα antibody (Santa Cruz, sc-365318, 5 μg), or the anti-IgG antibody (Cell Signaling Technology, 2729, 5 μg) as the control. DNA fragments were purified and used for qPCR using primers for mouse CD36 promoter or enhancer region containing C/EBPα or LXRβ binding sites. The primers used are listed in Table S2.

### Phagocytosis assays and SAB administration

Cells were seeded at a density of 2 × 10^5^ cells/well for 24 h before infection. Opsonized particles (latex beads) were made by being incubated with 50% mouse serum for 30 min at 37°C, or specific IgG at 1 mg/ml for 3 h. Cells were challenged with opsonized or unopsonized particles suspension (1:1,000) in DMEM containing 10% FBS or serum-free DMEM for 30 min at 37°C, washed with PBS, and fixed with 4% paraformaldehyde. Percentages of phagocytosis were determined by counting the cells including particles using OLYMPUS IX73 fluorescent microscope (Shinjuku, Tokyo, Japan) and Accuri C6 Flow Cytometry (BD Biosciences, San Jose, CA, USA).

Cells were seeded for 24 h before infection on a Lab-Tek chambered coverglass. The cells were incubated with *E. coli* K12 or UPEC strains at a multiplicity of infection (MOI) of 50 or 20 for 30 min at 37°C. Cells were then washed five times, fixed, and scored microscopically with a confocal fluorescence microscope (FV1000-D, Olympus, Tokyo, Japan). For inhibiting CD36 expression, different concentrations of SAB were added to RAW264.7 cells overnight before infection with *E. coli* strains. Percentages of phagocytosis were determined by counting the cells including bacteria.

### RNA extraction and real time RT-PCR

RNA of cells was isolated using the Total RNA Extraction Kit (Solarbio, Beijing, China) according to the manufacturer's protocol. RNA was converted to cDNA using the RevertAid First Strand cDNA Synthesis Kit (Thermo Scientific). The PCR reactions were performed with FastStart Universal SYBR Green Master mix (Roche) on a 7900 Fast Real-Time PCR System (Roch). The PCR conditions were 95°C for 5 min and 40 cycles of 95°C for 20 s, 60°C for 20 s, and 72°C for 20 s. β-actin was used as the endogenous control and data were normalized based on the transcription level of β-actin in the wild-type and then analyzed using the comparative critical threshold cycle 2^−ΔΔ*Ct*^ method. The primers used are listed in Table S2.

### Lentiviral production

The mouse CD36 gene was cloned into pCDH-CMV-MCS-EF1-copGFP. The shRNAs targeting CD36 were cloned into pLKO.1. The constructed plasmids, in addition with assistant vectors psPAX2 and pMD2.G, were transiently transfected into HEK293T cells. Viral supernatants were collected and used to infect RAW264.7 cells for 12 h. Lentivirus-transfected cells with stable GFP expression were sorted by FACSAria II Cell sorter (BD Biosciences), and lentivirus-transfected stable cells for shRNAs were selected using puromycin (10 μg/mL). The plasmids constructed and primers used are listed in Tables S1,S2.

### Flow cytometry analysis of cells

After treatment, RAW264.7 cells and mouse peritoneal macrophages were collected and washed twice with PBS, respectively. Cells (~1 × 10^6^) were treated with 1 μl anti-mouse CD36 polyclonal antibody conjugated to PE (Biolegend, San Diego, CA, USA). Cells treated with PE-conjugated rabbit IgG were used as the control. After 30 min of incubation on ice in the dark, cells were washed three times with PBS and determined by Accuri C6 Flow Cytometry (BD Biosciences).

### Mouse model of pyelonephritis

All animal experiments were performed according to the standards in the Guide for the Care and Use of Laboratory Animals (Institute of Laboratory Animal Resources of National Research Council, United States). All mouse studies were evaluated by the Animal Ethics Committee of Tianjin Medical University and Tianjin Institute of Pharmaceutical Research New Drug Evaluation Co. Ltd., (IACUC number: 2017082801), Tianjin, China. We made every effort to minimize animal suffering and to reduce animals used.

Acute UTIs in mice were established according to the previously described protocols for mouse model for pyelonephritis (35). UPEC strains were cultured overnight under static conditions in LB medium, harvested by centrifugation at 5000 × g for 10 min, and resuspended in PBS to a concentration of 2 × 10^10^ CFU/ml. Anesthetized female 6–8 week-old female C57BL/6J mice were infected by intraurethral inoculation of UPEC strains (10^9^ CFU for CFT073 and 10^8^ CFU for UTI89) two times at a 3 h interval. Mice were sacrificed at 24, 48, and 72 h after infections, the bladders and kidneys were removed, homogenized in 1 ml of PBS containing 0.025% Triton X-100, serially diluted and plated on LB agar plates for enumeration. The bladders and kidneys were also used for the following analysis.

### Flow cytometry analysis of tissues

The kidney and bladder tissues were sliced with a scalpel into many small pieces and digested for 30 min at 37°C with 0.5 mg/ml collagenase (Sigma-Aldrich) and 100 g/ml DNAse I in PBS. After digestion, tissues were mashed and single cell suspensions were filtered through a 70 μm nylon mesh and washed with PBS. Single cell suspensions were incubated with following antibodies: anti-CD11b conjugated to APC (eBioscience, San Diego, CA, USA), anti-Ly6G conjugated to PE (eBioscience). Cells were analyzed on a FACSCanto II Flow Cytometer (BD Biosciences) using the Flow Jo software (FlowJo, Ashland, OR, USA).

### Immunofluorescence analysis of tissues

The bladders and kidneys were aseptically harvested, and embedded in OCT compound with liquid nitrogen. Frozen sections (5 μm) were cut and air-dried at room temperature for 20 min and fixed with cold acetone for 10 min. Then the tissues were immediately submerged into methanol for 20 min and 3% hydrogen peroxide in methanol for 10 min. After rehydration in PBS, sections were blocked with 5% BSA for 1 h, incubated with F4/80 antibody (Abcam, ab6640, 1:100), CD36 antibody (Proteintech, 18836-1-AP, 1:100) in blocking buffer overnight at 4°C. After that, coverslips were washed five times with PBS, and incubated with FITC-labeled secondary antibody (Proteintech) or Alexa Fluor 488/594-labeled second antibody (Proteintech) for 1 h. Finally tissue sections were counterstained with DAPI for nuclei visualization. Images were acquired using the OLYMPUS IX73 fluorescent microscope (Shinjuku, Tokyo, Japan).

### H&E

The bladders and kidneys were fixed in 4% paraformaldehyde for 24 h and processed for paraffin embedding. Sections (5 μm) were used for hematoxylin and eosin (H&E) staining. Images were acquired using a microscope (Leica Microsystems, Wetzlar, Germany).

### Statistical analysis

Normality of distribution was analyzed by Shapito-Wilk test. When data have a normal distribution, statistical significance of differences between groups was calculated by two-tailed student's *t*-test, one-way ANOVA or two-way ANOVA analysis. For the analysis of bacterial titers and neutrophil percentages during UTIs, non-parametric Mann-Whitney test was used to calculate statistical significance.

## Results

### CNF1 promotes UPEC burden and increases neutrophils during acute UTIs using a pyelonephritis mouse model

**Figure 1:**
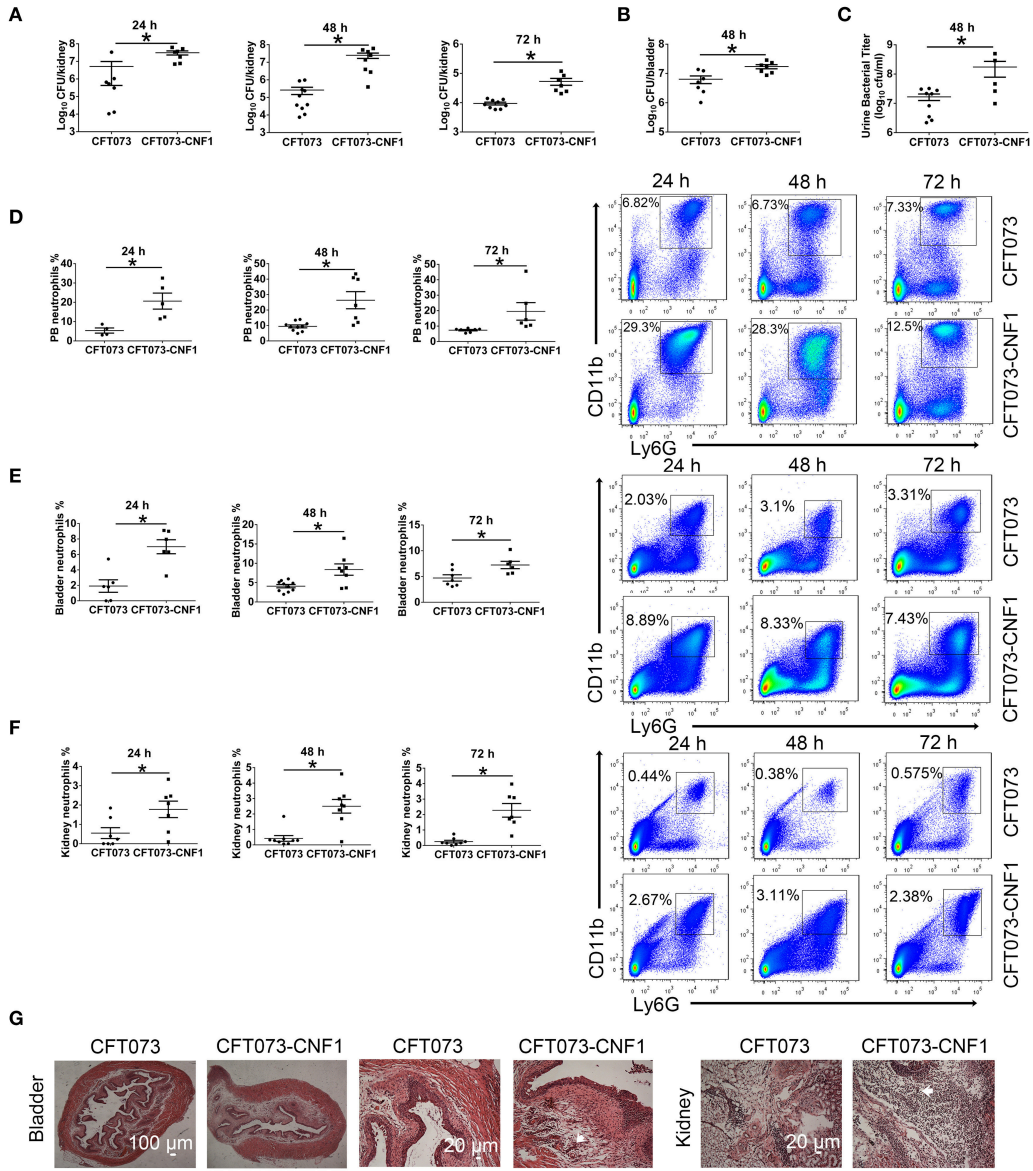
Effect of CNF1 on bacterial titers and neutrophil numbers during UTIs using CNF1-expressing CFT073 and vector control CFT073. Female C57BL/6J mice were transurethrally infected with 10^9^ CFU of CNF1-expressing CFT073 and vector control CFT073 two times at a 3 h interval, respectively. **(A–C)** Bacterial titers in kidney **(A)** were assessed at 24, 48, and 72 hpi, respectively (*n* = 7 to 10, two independent experiments). Bacterial titers in bladder **(B)** and urine **(C)** were assessed at 48 hpi (*n* = 5 to 9, two independent experiments). **(D–F)** Percentages of neutrophils in total cells in blood **(D)**, bladder **(E)** and kidney **(F)** were analyzed at 24, 48, and 72 hpi, respectively (*n* = 4 to 10, two independent experiments). Data are the mean ± SEM, non-parametric Mann-Whitney test, *P < 0.05. **(G)** Representative images of H&E staining of bladder and kidney tissues infected by CNF1-expressing CFT073 and vector control CFT073 at 48 hpi. The arrows indicate infiltrated neutrophils. Scale bar, 100 and 20 μm, respectively. The data from **(A)** and **(F)** are also present in Figures S1C,D.

To clarify the role of CNF1 in inflammation and bacterial clearance in kidney and bladder during acute UTIs, CNF1-expressing CFT073, and vector control CFT073, exhibiting similar growth rates (Figure S1A), were respectively used to transurethrally infect female C57BL/6J mice with 10^9^ CFU of strains twice at a 3 h interval. Bacterial titers at 24, 48, and 72 h post infection (hpi) in kidney tissues were obviously higher for CNF1-expressing CFT073, indicating that clearance of UPEC was much more effective in mice infected with vector control CFT073 compared with that in mice infected with CNF1-expressing CFT073 (Figure 1A). Significantly higher bacterial titers were also detected in bladder tissues and urine samples of mice infected with CNF1-expressing CFT073 at 48 hpi (Figures 1B,C). Obviously higher percentages of neutrophils in total cells were found in bladder, kidney and blood of mice infected with CNF1-expressing CFT073 at 24, 48, and 72 hpi, respectively (Figures 1D–F). More infiltrated neutrophils, serious edema, and urothelial damage were identified in bladder and kidney tissues infected with CNF1-expressing CFT073 by H&E staining (Figure 1G, Figure S1B).

**Figure 2:**
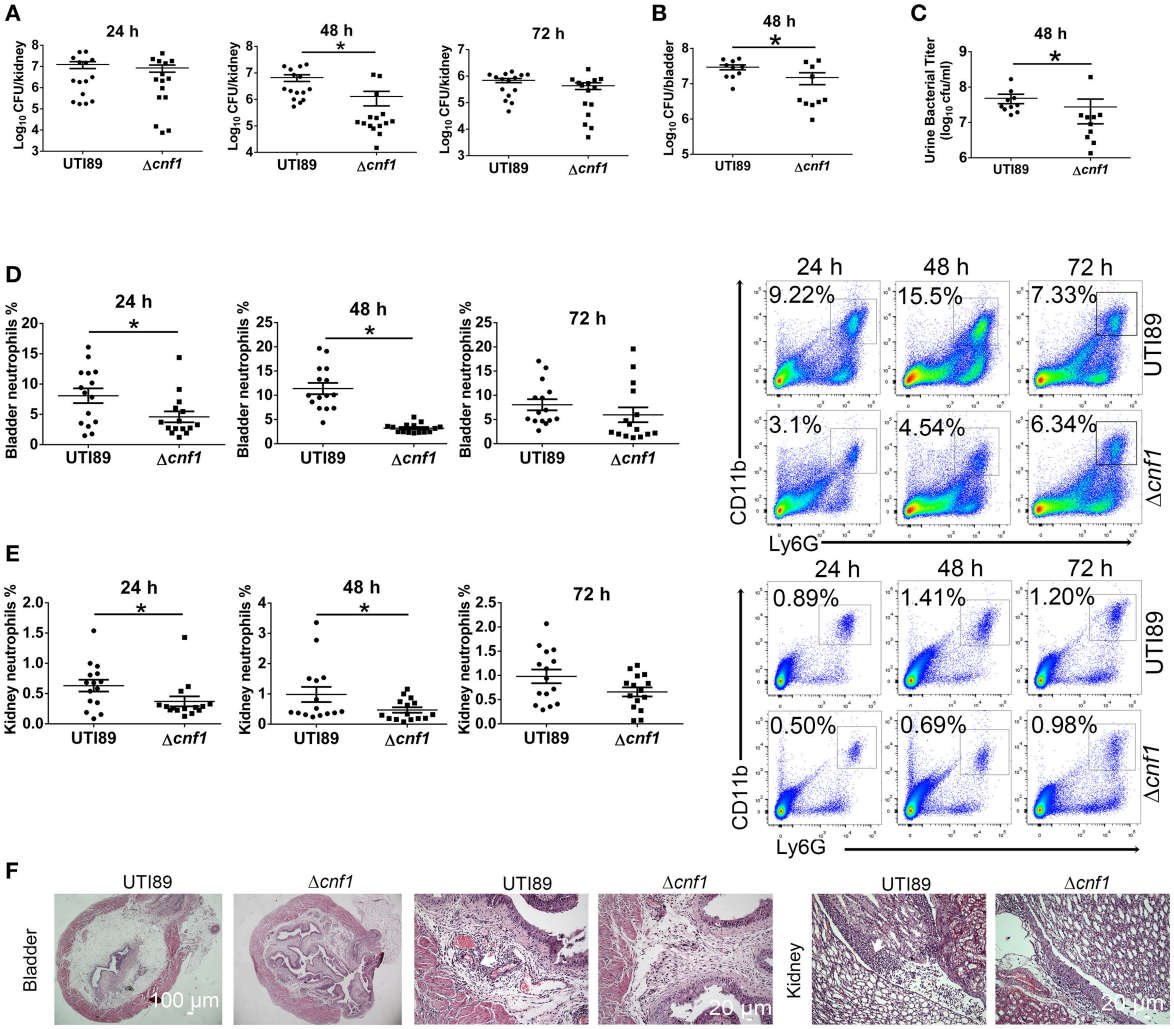
Effect of CNF1 on bacterial titers and neutrophil numbers during UTIs using UTI89 and Δcnf1. Female C57BL/6J mice were transurethrally infected with 10^8^ CFU of UTI89 and Δcnf1 two times at a 3 h interval, respectively. **(A–C)** Bacterial titers in kidney **(A)** were assessed at 24, 48, and 72 hpi, respectively (*n* = 15, three independent experiments). Bacterial titers in bladder **(B)** and urine **(C)** were assessed at 48 hpi (*n* = 10, two independent experiments). **(D,E)** Percentages of neutrophils in total cells in bladder **(B)** and kidney **(C)** were analyzed at 24, 48, and 72 hpi, respectively (*n* = 15, three independent experiments). Data are the mean ± SEM, non-parametric Mann-Whitney test, *P < 0.05. **(F)** Representative images of H&E staining of bladder and kidney tissues infected by UTI89 and Δcnf1 at 48 hpi. The arrows indicate infiltrated neutrophils. Scale bar, 100 and 20 μm, respectively.

UTI89 and a *cnf1* deletion strain derived from UTI89 (Δ*cnf1*), which exhibited similar growth rates (Figure S1A), were respectively used to transurethrally infect female C57BL/6J mice with 10^8^ CFU of strains twice at a 3 h interval. Bacterial titers at 48 hpi in kidney, bladder and urine were higher for UTI89 compared with that for Δ*cnf1* (Figures 2A–C). Higher percentages of neutrophils in total cells were found in bladder and kidney infected with the wild-type UTI89 at 24 and 48 hpi, and in blood at 48 hpi, respectively (Figures 2D,E, Figure S1E). There is a trend toward higher percentages of neutrophils for UTI89 comparing to Δ*cnf1* at 72 hpi with no statistical significance (Figures 2D,E). Infiltrated neutrophils, edema, and urothelial damage were more serious in bladder and kidney tissues infected with UTI89 compared with Δ*cnf1* by H&E staining (Figure 2F, Figure S1B).

As CNF1 seemed to play a role in recruiting much more neutrophils and inhibiting UPEC clearance, and macrophages are responsible for UPEC clearance in bladder and kidney (36–38), we wondered if CNF1 inhibited macrophage phagocytosis of UPEC. We also noted that, in kidney tissues of mice infected with vector control CFT073, neutrophils were decreased while bacteria were cleared from 24 to 72 h. However, neutrophils were not reduced when bacterial titers dropped from 48 to 72 h in the mice infected by CNF1-expressing CFT073 (Figures S1C,D). Thus, we proposed that CNF1 also impeded the removal of neutrophils from inflamed tissues.

### CNF1 reduces macrophage non-opsonic phagocytosis of UPEC *in vitro*

**Figure 3:**
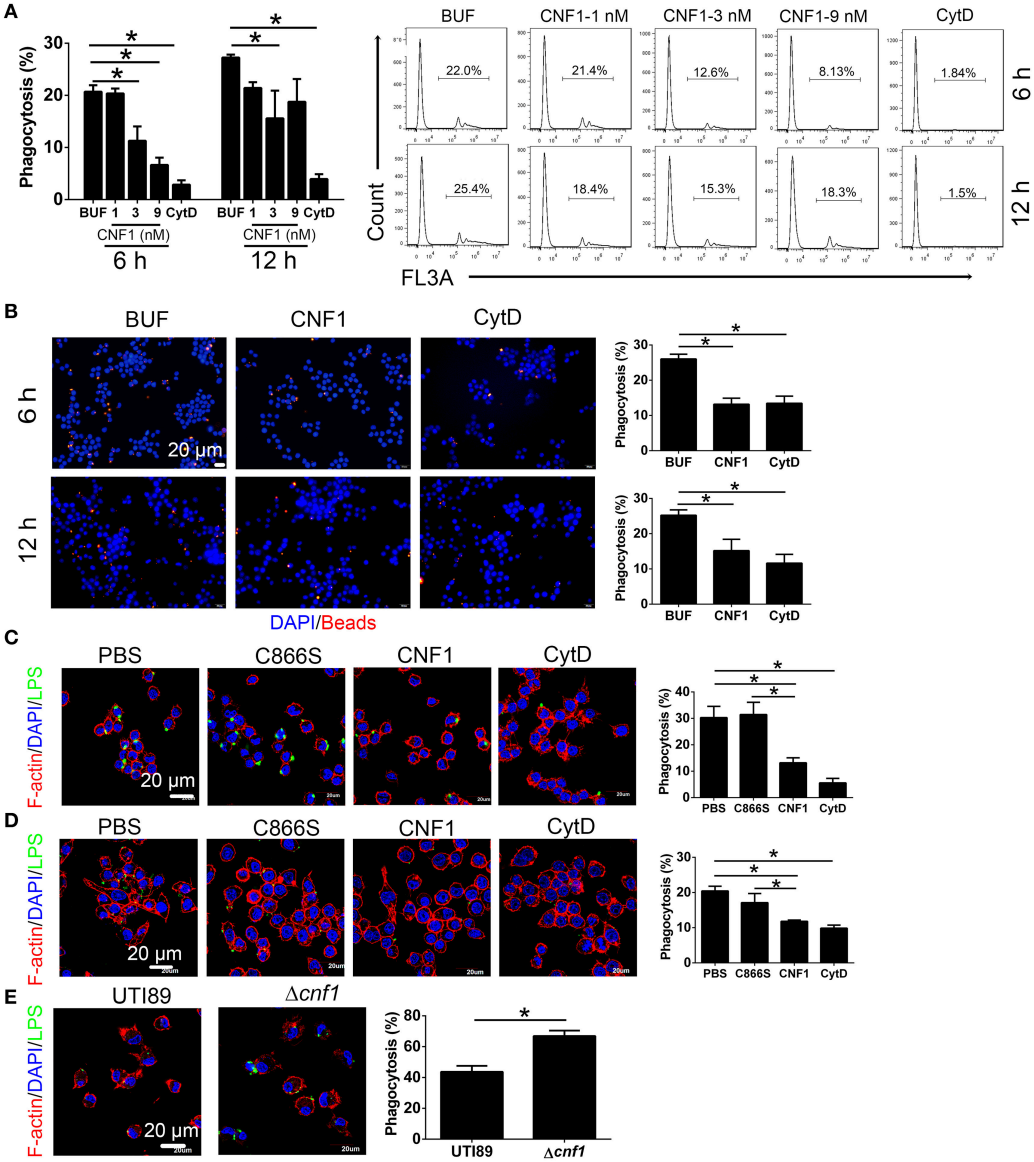
CNF1 reduces non-opsonic macrophage phagocytosis. RAW264.7 cells were treated with CNF1 in different doses, dialysis buffer, PBS, C866S (negative control), and CytD (5 μg/ml, positive control). **(A,B)** FACS **(A)** and immunofluorescence **(B)** analysis of phagocytosis of latex beads by RAW264.7 treated with different agents for 6 and 12 h. Blue, nucleus; red, latex beads **(C,D)** Immunofluorescence analysis of phagocytosis of *E. coli* K12 **(C)** and CFT073 **(D)** by RAW264.7 treated with different agents for 6 h. **(E)** Immunofluorescence analysis of phagocytosis of UTI89 and Δcnf1 by BMDMs. Scale bar, 20 μm. Blue, nucleus; red, F-actin; Green, LPS. Data are from three combined independent experiments **(A)**. 300 cells from three combined independent experiments each with two replicate wells **(B–D)**. Data are the mean ± SD, One-way ANOVA, *P < 0.05.

To determine the effect of CNF1 on macrophage phagocytosis, recombinant CNF1 was used to treat the murine macrophage cell line RAW264.7 for 6 h. Then the opsonic phagocytosis of iC3b- or IgG-opsonized latex beads and non-opsonic phagocytosis of non-opsonized latex beads were analyzed by flow cytometry. It was shown that CNF1 treatment did not affect opsonic phagocytosis by RAW264.7 (Figures S2A,B). However, CNF1 obviously reduced RAW264.7 phagocytosis of non-opsonized beads, as shown by flow cytometry and immunofluorescence assays (Figures 3A,B), and the inhibitory effect was shown in a dose-dependent manner (Figure 3A). The non-opsonized *E. coli* K12 or CFT073 strain was also used to validate the phenotype. CNF1 reduced RAW264.7 phagocytosis of *E. coli* K12 and CFT073 compared with CNF1-C866S (inactive) and PBS (Figures 3C,D). The effect of CNF1 on phagocytosis of *E. coli* K12 was evaluated using isolated mouse peritoneal macrophages and the macrophages derived from the human monocyte cell line THP-1 induced by PMA (Figures S2C,D). This effect was also validated using BMDMs infected by UTI89 and Δ*cnf1*, which showed that BMDM phagocytosis of UTI89 was lower than that of Δ*cnf1* (Figure 3E). CNF1 induced no cell death of RAW264.7 and BMDMs based on LDH assays (detecting release of lactate dehydrogenase, a cytoplasmic enzyme, into culture medium) (Figure S2E). These results indicate that CNF1 reduces UPEC clearance by inhibiting non-opsonic macrophage phagocytosis.

### CNF1 decreases CD36 expression in macrophages

**Figure 4:**
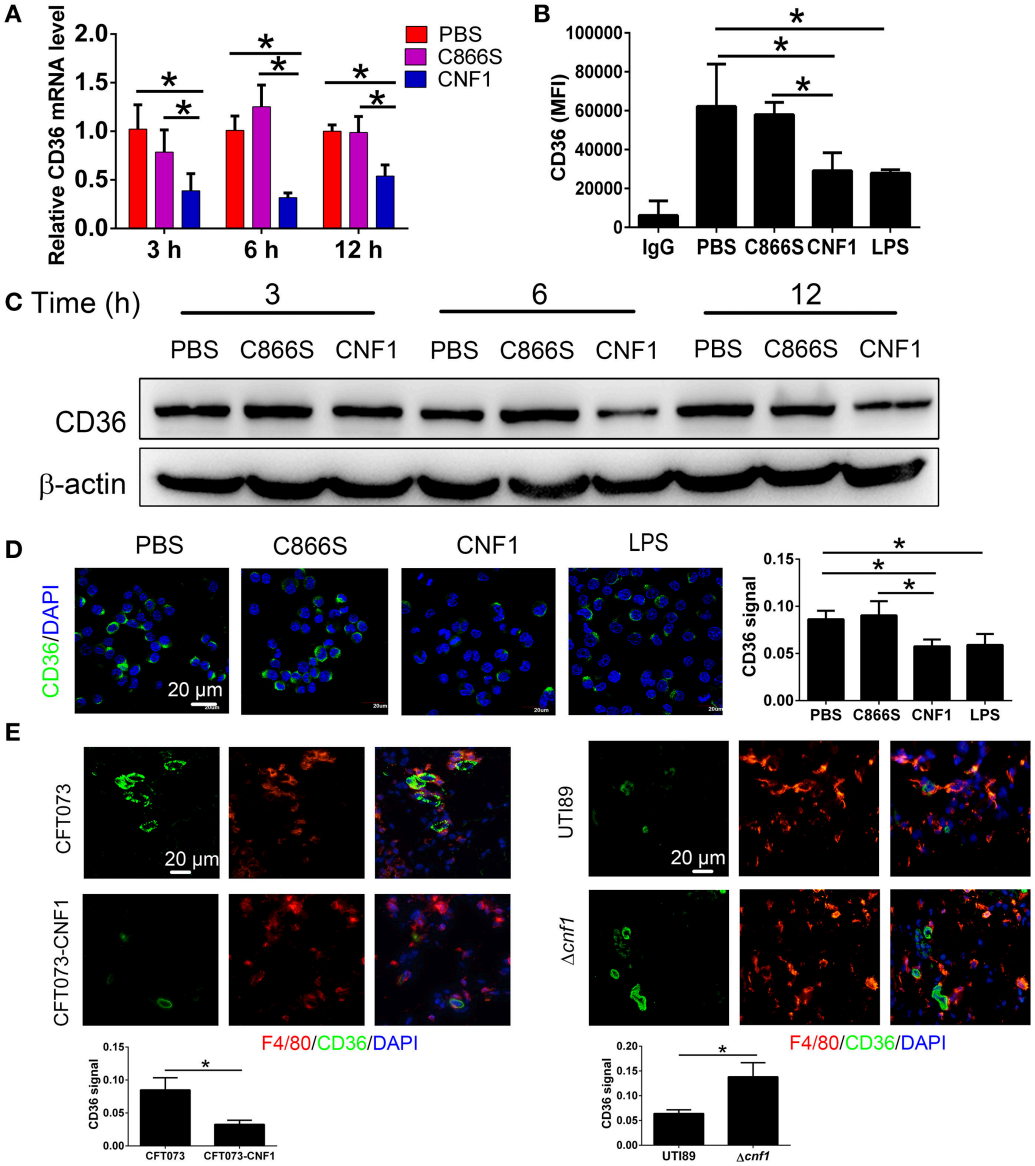
CNF1 reduces CD36 expression in RAW264.7. **(A)** qRT-PCR analysis of CD36 mRNA and protein levels in RAW264.7 treated with CNF1 (3 nM), C866S (3 nM), and PBS for 3, 6, and 12 h. **(B)** FACS analysis of CD36 expression in RAW264.7 treated with CNF1 (3 nM), C866S (3 nM), PBS and LPS (1 μg/ml, positive control) for 12 h. **(C)** Western blotting analysis of CD36 protein levels in RAW264.7 treated with CNF1 (3 nM), C866S (3 nM), and PBS for 3, 6, and 12 h. **(D)** Immunofluorescence analysis of CD36 expression in RAW264.7 treated with CNF1 (3 nM), C866S (3 nM), PBS, and LPS (1 μg/ml, positive control) for 6 h. Scale bar, 20 μm. **(E)** Immunofluorescence analysis of CD36 expression in macrophages of bladder tissues infected by CNF1-expressing CFT073 and vector control CFT073 at 24 hpi, and by UTI89 and Δcnf1 at 48 hpi, respectively. Scale bar, 20 μm. Blue, nucleus; red, F4/80; Green, CD36. Data are from three combined independent experiments each with two replicate wells (*n* = 6) **(A)**. Data are from four combined independent experiments **(B)**. Quantitative analysis of CD36 signal is using Image-Pro Plus, and data are from three combined independent experiments each with two fields (*n* = 6) **(D,E)**. Data are the mean ± SD, One-way ANOVA, *P < 0.05.

CD36 is a scavenger receptor of macrophages to mediate non-opsonic phagocytosis, and we proposed that CNF1 might modulate macrophage phagocytosis of UPEC by affecting CD36. We found both of CD36 mRNA and protein were reduced after treatment with CNF1 at 3, 6, and 12 h, compared with C866S and PBS, and decreased expression of CD36 by CNF1 was also detected in flow cytometry and immunofluorescence assays (Figures 4A–D). The effects of CNF1 on CD36 mRNA and protein were validated in BMDMs, mouse peritoneal macrophages, and THP-1 (Figures S3A–H). To determine whether CNF1 affects CD36 degradation, protein lysosomal, and proteasomal degradation were blocked using corresponding inhibitors (BafA1 and NH_4_CL for lysosomal degradation, MG132 for proteasomal degradation) during CNF1 treatment. Protein degradation inhibitors did not rescue CD36 expressing downregulated by CNF1 (Figure S3I). These findings demonstrate that CNF1 downregulated CD36 by affecting its transcription but not by inducing CD36 degradation. To further validate the effect of CNF1 on macrophage CD36 expression *in vivo*, we analyzed CD36 expression of macrophages in bladder tissues infected by CNF1-expressing CFT073, control vector CFT073, UTI89, and Δ*cnf1*, respectively. Macrophage CD36 expression was obviously reduced in CFT073-CNF1 and UTI89 infecting tissues compared with CFT073 and Δ*cnf1* by immunofluorescence assays (Figure 4E). As CD36 was important not only for bacterial clearance but also for apoptotic neutrophil elimination, these results suggest CNF1 contributes to increased bacterial titers and neutrophils during UPEC infections by decreasing CD36.

### Downregulation of CD36 inhibits non-opsonic macrophage phagocytosis

**Figure 5:**
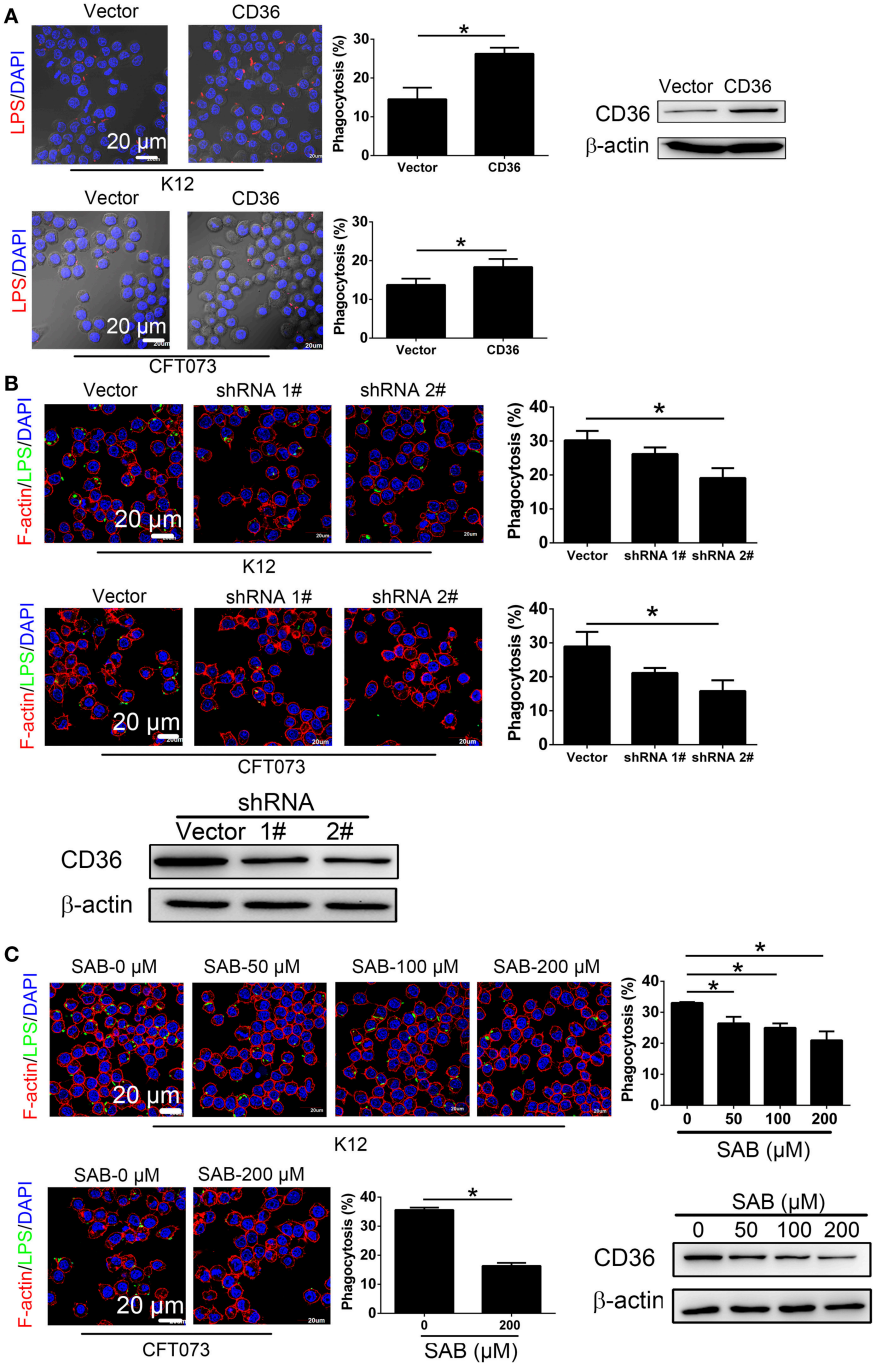
CD36 expression affects macrophage phagocytosis. **(A)** CD36 overexpression promotes RAW264.7 phagocytosis of *E. coli* K12 and CFT073 analyzed by immunofluorescence assays. Blue, nucleus; red, LPS. **(B)** CD36 knock-down reduces RAW264.7 phagocytosis of *E. coli* K12 and CFT073 analyzed by immunofluorescence assays. **(C)** CD36 inhibitor SAB reduces RAW264.7 phagocytosis of *E. coli* K12 and CFT073 analyzed by immunofluorescence assays. Scale bar, 20 μm. Blue, nucleus; red, F-actin; Green, LPS. 300 cells from three combined independent experiments each with two replicate wells **(A–C)**. Data are the mean ± SD, Student's *t*-test **(A)** or One-way ANOVA **(B,C)**, *P < 0.05.

To determine the effect of CD36 expression on macrophage phagocytosis of UPEC, RAW264.7 was transduced with constitutively expressed CD36, which was confirmed in the transduced cells (Figure 5A). Phagocytosis of non-opsonized *E. coli* K12 or CFT073 was increased in CD36 transduced cells (Figure 5A). CD36 expression in RAW264.7 was knocked down using shRNAs, and phagocytosis of *E. coli* K12 or CFT073 was reduced in knocked-down cells (Figure 5B). We also reduced CD36 expression using salcianolic acid B (SAB), an effective CD36 inhibitor (39). Percentages of macrophage phagocytosis were significantly attenuated in SAB dose-dependent manner (Figure 5C). Taken together, these results provide strong evidence that CNF1 reduces non-opsonic macrophage phagocytosis by downregulating transcription of CD36.

### CNF1 decreases CD36 transcription via C/EBPα and LXRβ

**Figure 6:**
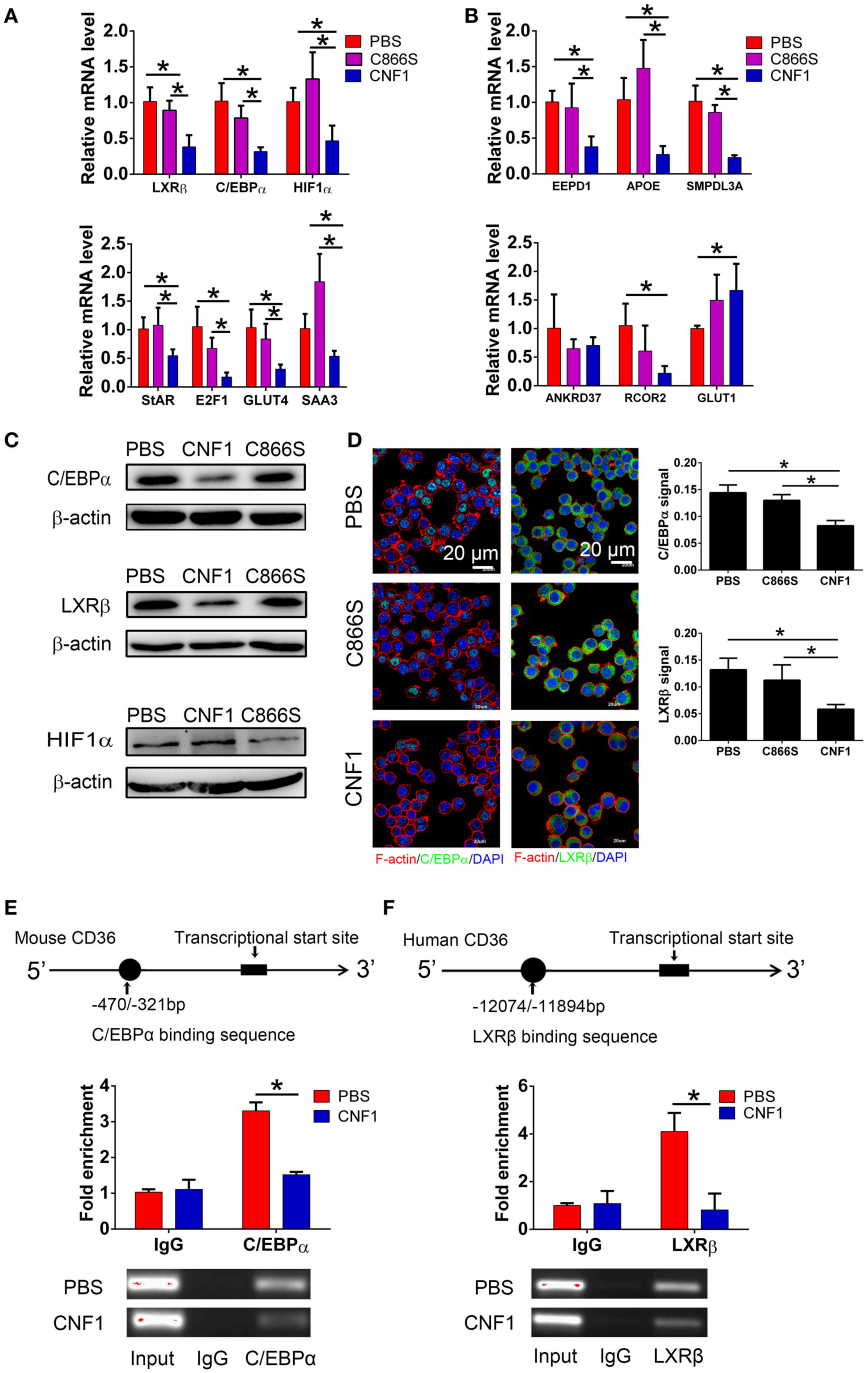
CNF1 attenuates CD36 expression by decreasing LXRβ and C/EBPα expressions and their recruitment to the CD36 promotor. **(A,B)** qRT-PCR analysis of mRNA levels for LXRβ, C/EBPα, HIF1α **(A)** and genes regulated by LXRβ, C/EBPα, and HIF1α **(B)** in RAW264.7 treated with CNF1 (3 nM), C866S (3 nM) and PBS for 6 h. **(C)** Western blotting analysis of protein levels for C/EBPα, LXRβ, and HIF1α in RAW264.7 treated with CNF1 (3 nM), C866S (3 nM), and PBS for 6 h. **(D)** Immunofluorescence analysis of C/EBPα and LXRβ expressions in RAW264.7 treated with CNF1 (3 nM), C866S (3 nM), and PBS for 6 h. Scale bar, 20 μm. Blue, nucleus; red, F-actin; Green, C/EBPα or LXRβ. **(E,F)** The binding of C/EBPα to the CD36 promoter in RAW264.7 **(E)** and LXRβ to the CD36 promoter in THP-1 **(F)** with or without CNF1 treatment (3 nM, 6 h) by ChIP-qPCR analysis. The enrichment is calculated by normalizing to nonspecific IgG in each sample. Data are from three combined independent experiments each with two replicate wells (*n* = 6) **(A,B)**. Quantitative analysis of C/EBPα and LXRβ signal is using Image-Pro Plus, and data are from three combined independent experiments each with two fields (*n* = 6) **(D)**. Data are from three combined independent experiments **(E,F)**. Data are the mean ± SD, One-way ANOVA, *P < 0.05.

As CD36 was reported to be transcriptionally regulated by several nuclear receptors such as liver X receptor (LXR), pregnane X receptor (PXR), peroxisome proliferator-activated receptor gamma (PPARγ) (40), farnesoid X receptor (FXR) (41), aryl hydrocarbon receptor (AHR) (42), and transcription factors such as CCAAT/enhancer-binding protein (C/EBP) (43) and hypoxia-inducible factor-1 (HIF-1) (44), we analyzed the effect of CNF1 on expressions of these factors. First, we identified the mRNA levels of these factors in RAW264.7 by qRT-PCR. The mRNA expressions of only three factors including LXRβ, C/EBPα, and HIF-1α could be detected, and expressions of other factors were too low to be detected (Figure S4A). Thus, the effects of CNF1 on these three factors were further evaluated. LXRβ and C/EBPα mRNA levels in RAW264.7 were significantly reduced by CNF1 at 3 h, 6 h, and 12 h, compared to C866S and PBS, while HIF-1α mRNA level was only significantly reduced by CNF1 at 6 h (Figure 6A, Figure S4B). We further examined the effect of CNF1 on expressions of genes regulated by LXRβ, C/EBPα, and HIF-1α, respectively. It was shown that, mRNA levels of the genes regulated by LXRβ or C/EBPα were also significantly reduced by CNF1. However, CNF1 did not reduced mRNA levels of genes regulated by HIF-1α (Figure 6B). The CNF1's effects on mRNA levels of LXRβ and C/EBPα and their downstream genes were also validated in mouse peritoneal macrophages, THP-1, and BMDMs, which were corresponding to the effect tested in RAW264.7 (Figures S4C–E). Protein levels of LXRβ and C/EBPα were also decreased by CNF1 compared with treatment by C866S and PBS using western blotting and immunofluorescence assays (Figures 6C,D). The same effects were validated in mouse peritoneal macrophages and THP-1 (Figures S4F–I).

To determine whether recruitment of LXRβ and C/EBPα to CD36 promoter was affected by CNF1, a ChIP assay was performed. C/EBPα has been identified to bind to nucleotides −470 to −321 of the CD36 promotor in mouse cells (43). Binding of C/EBPα to the same element of the CD36 promotor was detected in RAW264.7, and CNF1 treatment dramatically decreased recruitment of C/EBPα to CD36 promotor (Figure 6E). LXRα and LXRβ share a high degree of similarity about 80% based on amino acids, and are considered paralogues (45). LXRα has been identified to bind to nucleotides −12074 to −11894 and −3126 to −2945 of the CD36 promotor in human cells based on ChIP-seq experiments (46). We proposed LXRβ was recruited to the same elements within the two locations. However, only binding of LXRβ to nucleotides −12074 to −11894 were detected in PMA-stimulated THP-1 cells (Figure S4J). We further determined that recruitment of LXRβ to nucleotides −12074 to −11894 of CD36 promotor was reduced by CNF1 (Figure 6F). Taken together, these findings demonstrate that CNF1 attenuates CD36 expression by decreasing expressions of its upstream transcription factors LXRβ and C/EBPα and inhibiting their recruitment to the CD36 promotor.

### CNF1 inhibits LXRβ expression through activating Cdc42

**Figure 7:**
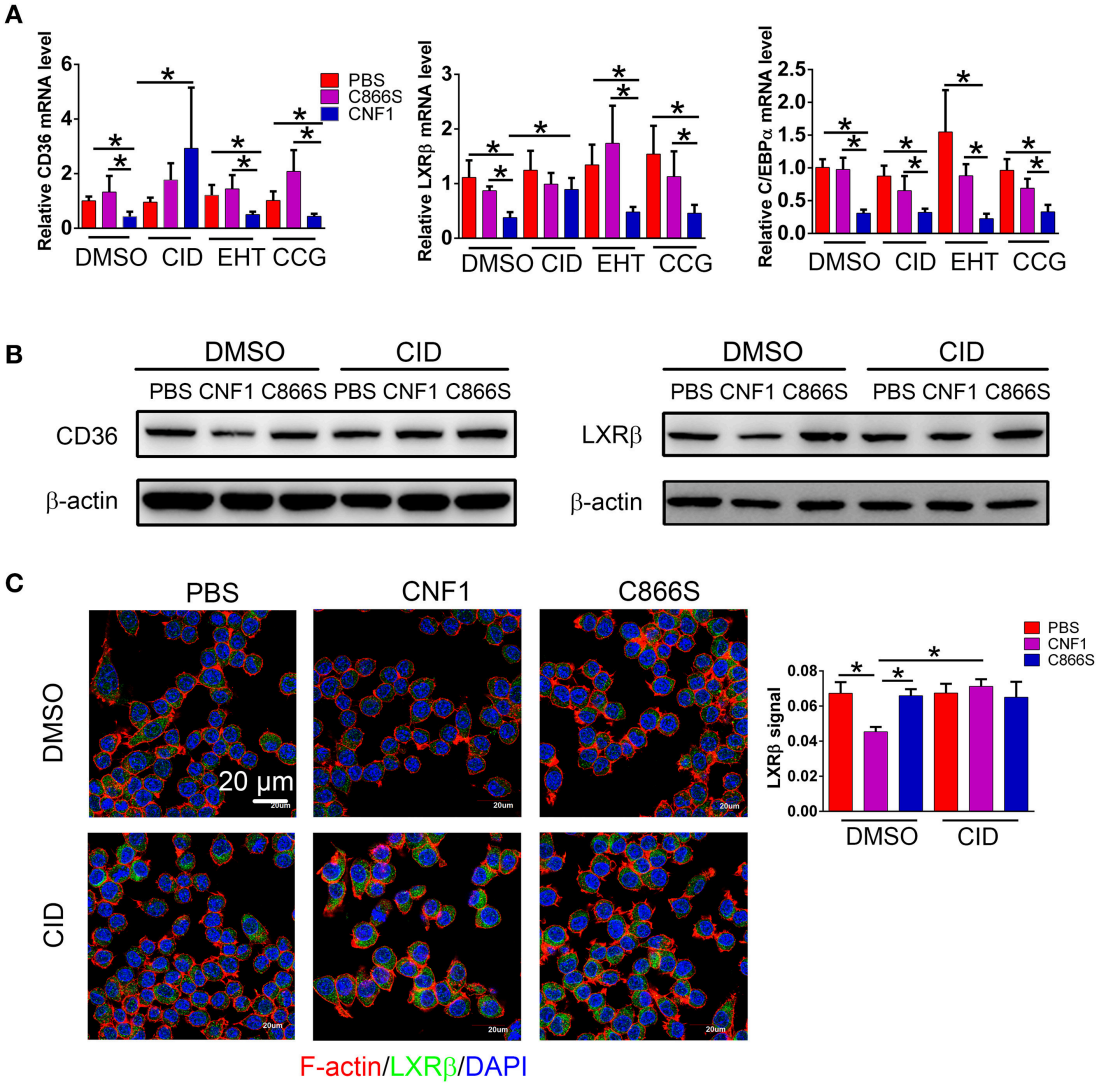
CNF1 affects CD36 expression partially through Cdc42-LXRβ signaling axis. **(A)** qRT-PCR analysis of mRNA levels for CD36, LXRβ, and C/EBPα in RAW264.7 treated with CNF1 (3 nM), C866S (3 nM), and PBS as well as the Rac1 inhibitor EHT 1864 (EHT, 25 μM), the Cdc42 inhibitor CID44216842 (CID, 20 μM), the RhoA inhibitor CCG-1423 (CCG, 20 μM) for 12 h, respectively. **(B)** Western blotting analysis of CD36 and LXRβ expressions in RAW264.7 treated with CNF1 (3 nM), C866S (3 nM), and PBS as well as the Cdc42 inhibitor (CID, 20 μM) for 12 h, respectively. **(C)** Immunofluorescence analysis of LXRβ expression in RAW264.7 treated with CNF1 (3 nM), C866S (3 nM), and PBS as well as the Cdc42 inhibitor (CID, 20 μM) for 12 h. Scale bar, 20 μm. Blue, nucleus; red, F-actin; Green, LXRβ. Data are from three combined independent experiments each with two replicate wells (*n* = 6) **(A)**, Quantitative analysis of LXRβ signal is using Image-Pro Plus, and data are from three combined independent experiments each with two fields (*n* = 6) **(C)**. Data are the mean ± SD, Two-way ANOVA, *P < 0.05.

We previously reported that CNF1 could enter and translocate into cytoplasm of epithelial cells such as PC3. However, its location and protein interaction profile in macrophages were not determined. The presence of CNF1 was detected in RAW264.7 after treatment for 6 h by western blotting (Figure S5A), and three Rho GTPases including RhoA, Cdc42, and Rac1 were found to be activated by CNF1 using a pull-down assay (Figure S5B). To examine which Rho GTPase is involved in regulation of LXRβ, C/EBPα, and CD36 by CNF1, we blocked the three GTPases using specific inhibitors. While CD36 and LXRβ mRNA reduced by CNF1 were rescued by the Cdc42 inhibitor CID44216842, C/EBPα mRNA level was not rescued by any of the inhibitors for the three Rho GTPases (Figure 7A). The Cdc42 inhibitor was also identified to rescue CD36 and LXRβ, but not C/EBPα mRNA expressions decreased by CNF1 in THP-1 cells (Figure S5C). We further examined the effect of Cdc42 on CD36 and LXRβ protein levels using western blotting and immunofluorescence assays, and found that the Cdc42 inhibitor blocked CNF1's regulation of CD36 and LXRβ expressions (Figures 7B,C). These results suggest CNF1 partially inhibits CD36 expression through the Cdc42-LXRβ signaling axis.

## Discussion

Macrophages have been reported to be responsible for UPEC phagocytosis in bladder and kidney (36–38), and CNF1 has been reported to cause apoptosis of bladder cells, possibly leading to bladder cell exfoliation and bacterial access to underlying tissues to interact with macrophages (8, 12, 47). In this study, we investigated that phagocytosis of UPEC by macrophages was reduced by CNF1.

**Figure 8:**
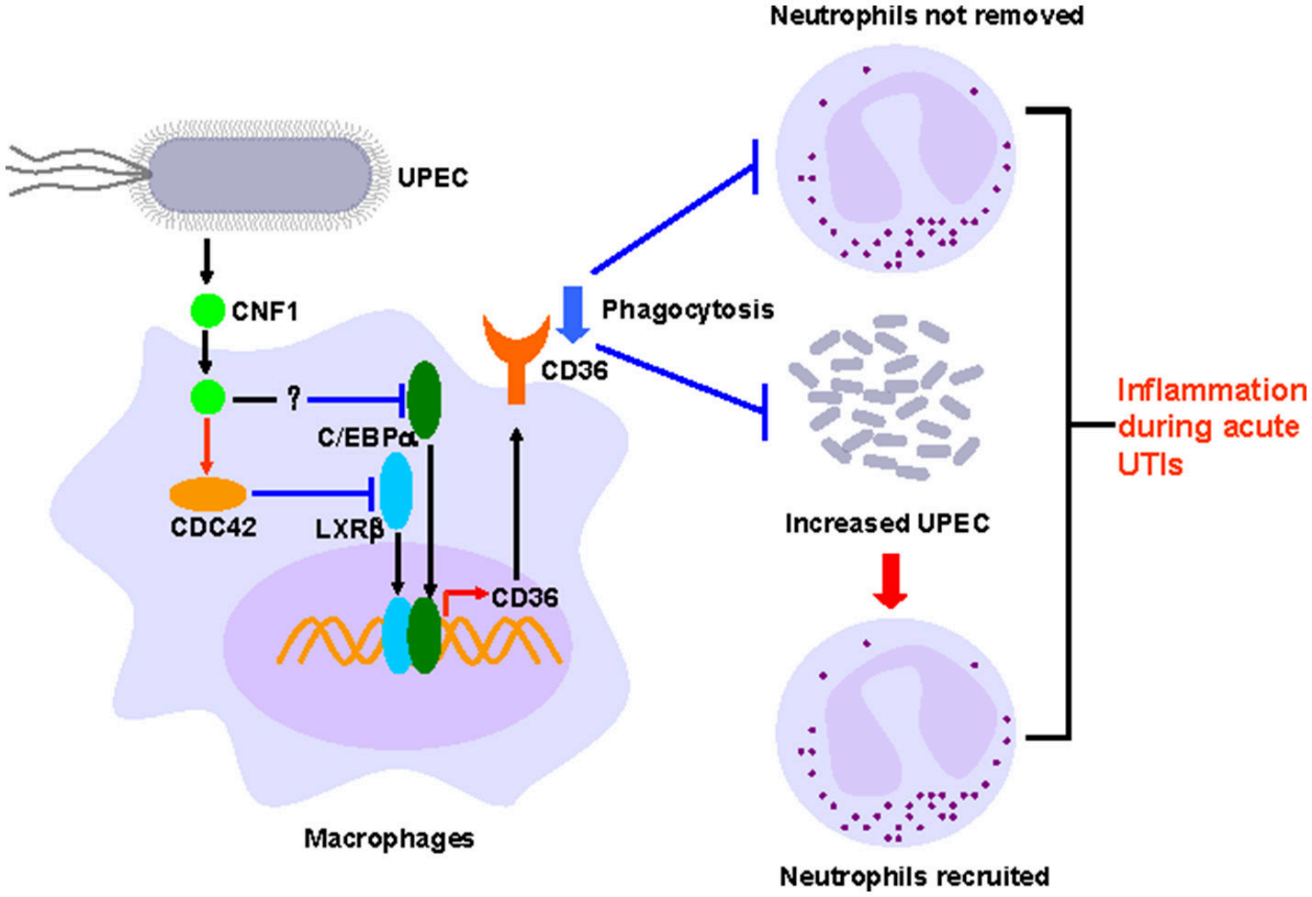
Model of CNF1-mediated downregulation of CD36 in macrophages, which affects host innate immune defense and homeostasis during acute UTIs.

The most well studied effect of CNF1 from UPEC on host cells is that CNF1 could accelerate bacterial invasion, and the detailed mechanism has also been clarified (22–24, 25). However, the effect of CNF1 on proteins downstream of Rho GTPases is limited. In our previous study, we reported that CNF1 induced the migration and invasion of prostate cancer cells to promote prostate cancer progression by activating the Cdc42-PAK1 axis (26). In this study, we found that CNF1 downregulated the transcription of CD36 partially depending on Cdc42. The transcription of CD36 is regulated by several transcriptional factors in different kinds of cells and pathological processes. We found CNF1 downregulated CD36 transcription by decreasing the expressions of C/EBPα and LXRβ and their binding to the CD36 promotor, which clarified the signal pathway downstream of Rho GTPases to affect CD36. Although CD36 is well-known for its functions in pathogens and apoptotic neutrophils clearance, and has been reported to be essential to control the host innate response to *Staphylococcus aureus* skin infections (48), negative regulation of CD36 by bacteria or bacterial toxins has not been investigated. We demonstrated that CNF1 decreased CD36 expression, thus attenuating UPEC and neutrophil clearance, resulting in severe inflammation during acute UTIs (Figure 8). Therefore, CD36 has the potential to be used as a possible target for acute UTI therapy because of its effect on host defenses and homeostasis.

The roles of CNF1 in bacterial clearance and inflammation during acute UTIs are not consistent. Some studies found that CNF1 from a clinical isolated UPEC strain CP9 induced inflammation and tissue damage in bladder, kidney, and prostate (27–29), and Michaud reported that CNF1 from a clinical isolated UPEC strain U8 and a *E. coli* strain RS218 isolated from a patient with meningitis had no effect on inflammation in bladder and kidney (32). For bacterial clearance, Rippere-Lampe reported that CNF1 increased UPEC colonization in bladder and kidney using UPEC clinical strains CP9 and C189 (30); whereas, Michaud reported that CNF1 had no effect on bacteria colonization using U8 and RS218 (32); and Diabate reported that CNF1 decreased UPEC in blood while inducing inflammation using UTI89Δ*hlyA* strain in a bacteremia mouse model (31). In our study, two widely reported UPEC strains UTI89 (comparing to UTI89Δ*cnf1*) and CFT073 (comparing to CNF1-expressing CFT073) were used to transurethrally infect C57BL/6J mice using a pyelonephritis mouse model (35, 38), and found that CNF1 decreased UPEC clearance and increased neutrophil infiltration and tissue damage in bladder and kidney. Based on these published papers and our study, we hypothesized that the effect of CNF1 on bacterial clearance and inflammation were affected by four factors. (1) The type of UPEC strain: CNF1 from most UPEC clinical strains except U8 induced inflammation according to our study and others (27–31). (2) The bacterial infection dosage: 10^7^ to 2 × 10^9^ CFU has been used in urinary tract infection mouse models (49–52), and a high dosage is necessary for kidney infection and CNF1's effect on bacterial clearance (30, 35, 38). In this study, CNF1's effect on bacterial clearance is more obvious by using 10^9^ CFU of CFT073 than 10^8^ CFU of UTI89. (3) The type of mouse: different mice have different responses to UPEC infections. Therefore, the inoculation dosage should be different (30, 53). (4) The type of tissue: bladder is easily to be colonized by UPEC at a low dosage, whereas kidney infection requires a high dosage (35, 38).

In this study, the role of CNF1 in host inflammation and bacterial clearance during acute UTIs using a pyelonephritis mouse model were identified and the mechanism was clarified by *in vitro* experiments.

## Author contributions

QW designed the study. HY, QL, CW, JW, JL, and LW performed the majority of experiments. QW, HY, Z-SZ, and ZY analyzed the data and wrote the paper. All authors discussed the data and reviewed the manuscript.

### Conflict of interest statement

The authors declare that the research was conducted in the absence of any commercial or financial relationships that could be construed as a potential conflict of interest.

## Supplementary material

Figure S1 CNF1 increases bacteria titers and neutrophil numbers in kidney. **(A)** Growth curves of CNF1-expressing CFT073, vector control CFT073, UTI89, and Δ*cnf1*. **(B)** General morphology of bladder and kidney infected by CNF1-expressing CFT073, vector control CFT073, UTI89, and Δ*cnf1* at 48 hpi. **(C)** Bacteria titers in kidney infected by CNF1-expressing CFT073 and vector control CFT073 at 24, 48, and 72 hpi, respectively. **(D)** Percentages of neutrophils in total cells in kidney infected by CNF1-expressing CFT073 and vector control CFT073 at 24, 48, and 72 hpi, respectively. The data present in **(C,D)** are from Figures 1A,F. **(E)** Percentages of neutrophils in total cells in blood were analyzed at 48 hpi (*n* = 10, two independent experiments). Data are the mean ± SEM. nonparametric Mann–Whitney test, ^*^*P* < 0.05.

Figure S2 CNF1 does not reduces opsonic phagocytosis in macrophages. **(A,B)** FACS analysis of phagocytosis of IgG-opsonized **(A)** and iC3b-opsonized **(B)** latex beads by RAW264.7 treated with CNF1 (3 nM) and dialysis buffer for 6 h. **(C,D)** Immunofluorescence analysis of phagocytosis of *E. coli* K12 by mouse peritoneal macrophages (PM) **(C)** and THP-1 **(D)** treated with CNF1 (3 nM), PBS, C866S (3 nM, negative control), and CytD (5 μg/ml, positive control) for 6 h. Scale bar, 20 μm. Blue, nucleus; red, F-actin; Green, LPS. Bar graphs represent data from at least three independent experiments. **(E)** LDH assays of RAW264.7 and BMDMs treated with CNF1 (3 nM), PBS, C866S (3 nM) for 6 and 12 h. Data are from three combined independent experiments **(A,B)**. Three hundred cells from three combined independent experiments each with two replicate wells **(C,D)**. Data are from four combined independent experiments **(E)**. Data are the mean ± SD, One-way ANOVA, ^*^*P* < 0.05.

Figure S3 CNF1 reduces CD36 expression in PM and THP-1. **(A–C)** qRT-PCR **(A)**, FACS **(B)**, and western blotting **(C)** analysis of CD36 mRNA level in BMDMs treated with CNF1 (3 nM), C866S (3 nM), and PBS for 6 h. **(D)** qRT-PCR analysis of CD36 mRNA level in PM treated with CNF1 (3 nM), C866S (3 nM), and PBS for 6 h. **(E)** FACS analysis of CD36 expression in PM treated by CNF1 (3 nM), C866S (3 nM), PBS, and LPS (1 μg/ml, positive control) for 12 h. **(F,G)** Immunofluorescence analysis of CD36 expression in PM **(D)** and THP-1 **(E)** treated by CNF1 (3 nM), C866S (3 nM), PBS and LPS (1 μg/ml, positive control) for 6 h. Scale bar, 20 μm. Blue, nucleus; Green, CD36. **(H)** qRT-PCR analysis of CD36 mRNA level in THP-1 treated with CNF1 (3 nM), C866S (3 nM), and PBS for 6 h. **(I)** Western blotting analysis of CD36 protein level in RAW264.7 treated with CNF1 as well as lysosomal and proteasomal degradation inhibitors for 12 h (BafA1 and NH_4_CL for lysosomal degradation, MG132 for proteasomal degradation). Data are from three combined independent experiments each with two replicate wells (*n* = 6) **(A,D,H)**. Data are from four combined independent experiments **(B,E)**. Quantitative analysis of CD36 signal is using Image-Pro Plus, and data are from three combined independent experiments each with two fields (*n* = 6) **(F,G)**. Data are the mean ± SD, One-way ANOVA, ^*^*P* < 0.05.

Figure S4 CNF1 attenuates CD36 expression by decreasing LXRβ and C/EBPα expressions in PM and THP-1. **(A)** qRT-PCR analysis of mRNA levels for upstream transcriptional regulators of CD36 in RAW264.7. **(B)** qRT-PCR analysis of mRNA levels for LXRβ, C/EBPα, HIF1α in RAW264.7 treated with CNF1 (3 nM), C866S (3 nM) and PBS for 3 and 12 h, respectively. **(C,D)** qRT-PCR analysis of mRNA levels for CD36, LXRβ, C/EBPα, HIF1α and genes regulated by LXRβ and C/EBPα in PM **(C)** and THP-1 **(D)** treated with CNF1 (3 nM), C866S (3 nM), and PBS for 6 h. **(E)** qRT-PCR analysis of mRNA levels for LXRβ and C/EBPα, HIF1α in BMDMs treated with CNF1 (3 nM), C866S (3 nM) and PBS for 6 h. **(F–G)** Western blotting analysis of protein levels for C/EBPα and LXRβ in PM **(F)** and C/EBPα in THP-1 **(G)** treated with CNF1 (3 nM), C866S (3 nM), and PBS for 6 h. **(H–I)** Immunofluorescence analysis of C/EBPα and LXRβ expressions in PM **(H)** and THP-1 **(I)** treated with CNF1 (3 nM), C866S (3 nM) and PBS for 6 h. Scale bar, 20 μm. Blue, nucleus; red, F-actin; Green, C/EBPα or LXRβ. **(J)** The binding of LXRβ to the two sites of the CD36 promoter in THP-1 by ChIP-qPCR analysis. Data are from three combined independent experiments each with two replicate wells (*n* = 6) **(A–E)**. Quantitative analysis of C/EBPα and LXRβ signal is using Image-Pro Plus, and data are from three combined independent experiments each with two fields (*n* = 6) **(H–I)**. Data are from three combined independent experiments **(J)**. Data are the mean ± SD, One-way ANOVA, ^*^*P* < 0.05.

Figure S5 CNF1 activates Rho GTPases in macrophages and afffects LXRβ through Cdc42 in THP-1. **(A)** Western blotting analysis of RAW264.7 cells cultured with the CNF1 (3 nM) for 6 h. **(B)** Western blotting analysis of activated Rho GTPases including RhoA, Cdc42, and Rac1 after immunoprecipitation with GTP-pull down assays using specific antibodies. **(C)** qRT-PCR analysis of mRNA levels for CD36, LXRβ, and C/EBPα in THP-1 treated with CNF1 (3 nM), C866S (3 nM) and PBS as well as the Rac1 inhibitor EHT 1864 (EHT, 25 μM), the Cdc42 inhibitor CID44216842 (CID, 20 μM), the RhoA inhibitor CCG-1423 (CCG, 20 μM) for 12 h, respectively. Bar graphs represent data from at least three independent experiments. Data are from three combined independent experiments each with two replicate wells (*n* = 6) **(C)**. Data are the mean ± SD, Two-way ANOVA, ^*^*P* < 0.05.

Table S1 Strains and plasmids in this study.

Table S2 Primers used in this study.
