# Supplementary figures and images for: Cytotoxic Necrotizing Factor 1 Downregulates CD36 Transcription in Macrophages to Induce Inflammation During Acute Urinary Tract Infections
Source: Front Immunol. 2018 Aug 31;9:1987. doi: 10.3389/fimmu.2018.01987 (PMC6128224; doi:10.3389/fimmu.2018.01987)

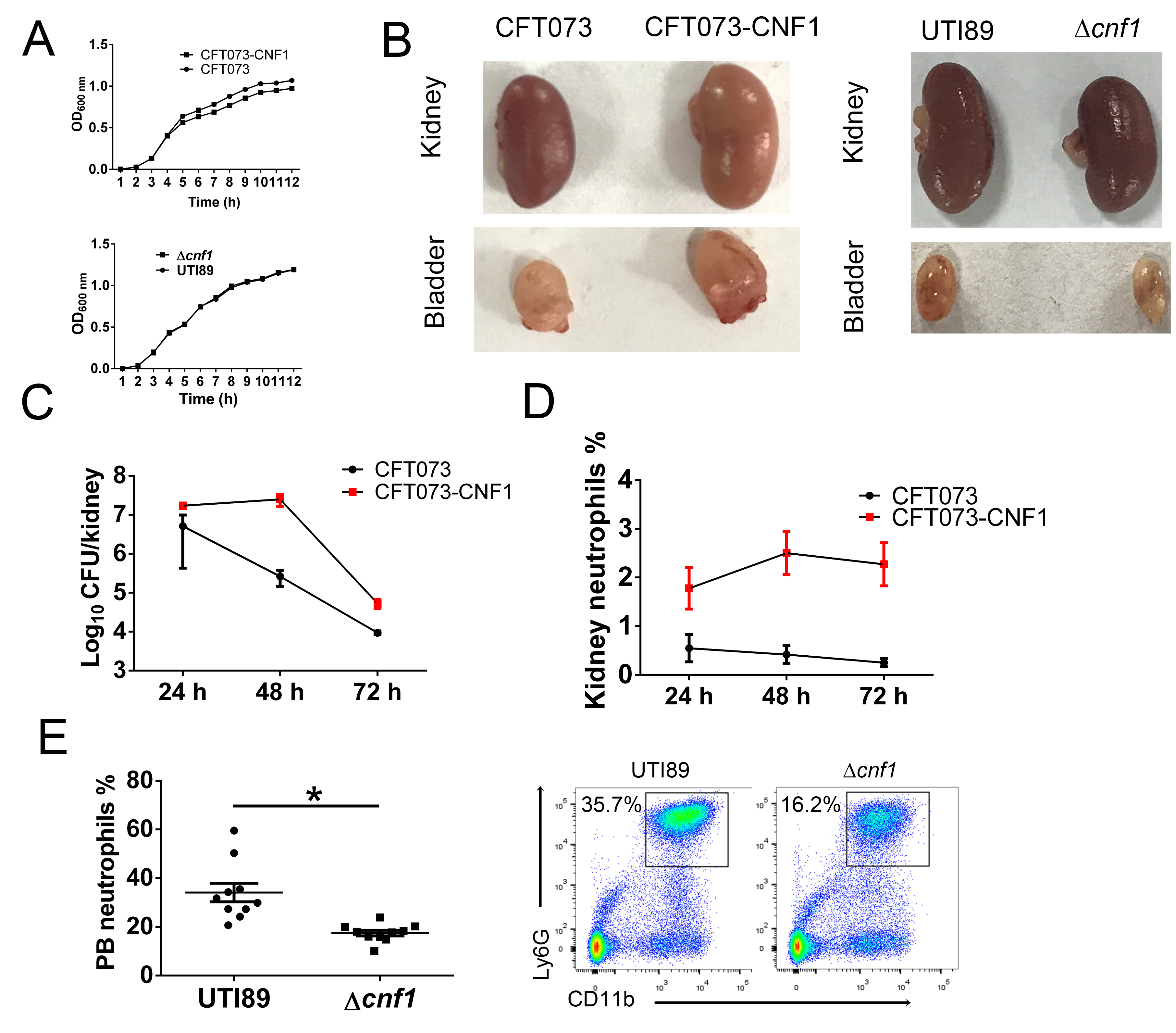

Supplement: Figure S1 — CNF1 increases bacteria titers and neutrophil numbers in kidney. (A) Growth curves of CNF1-expressing CFT073, vector control CFT073, UTI89, and Δcnf1. (B) General morphology of bladder and kidney infected by CNF1-expressing CFT073, vector control CFT073, UTI89, and Δcnf1 at 48 hpi. (C) Bacteria titers in kidney infected by CNF1-expressing CFT073 and vector control CFT073 at 24, 48, and 72 hpi, respectively. (D) Percentages of neutrophils in total cells in kidney infected by CNF1-expressing CFT073 and vector control CFT073 at 24, 48, and 72 hpi, respectively. The data present in (C,D) are from Figures 1A,F. (E) Percentages of neutrophils in total cells in blood were analyzed at 48 hpi (n = 10, two independent experiments). Data are the mean ± SEM. nonparametric Mann–Whitney test, *P < 0.05. [file Image_1.TIF]

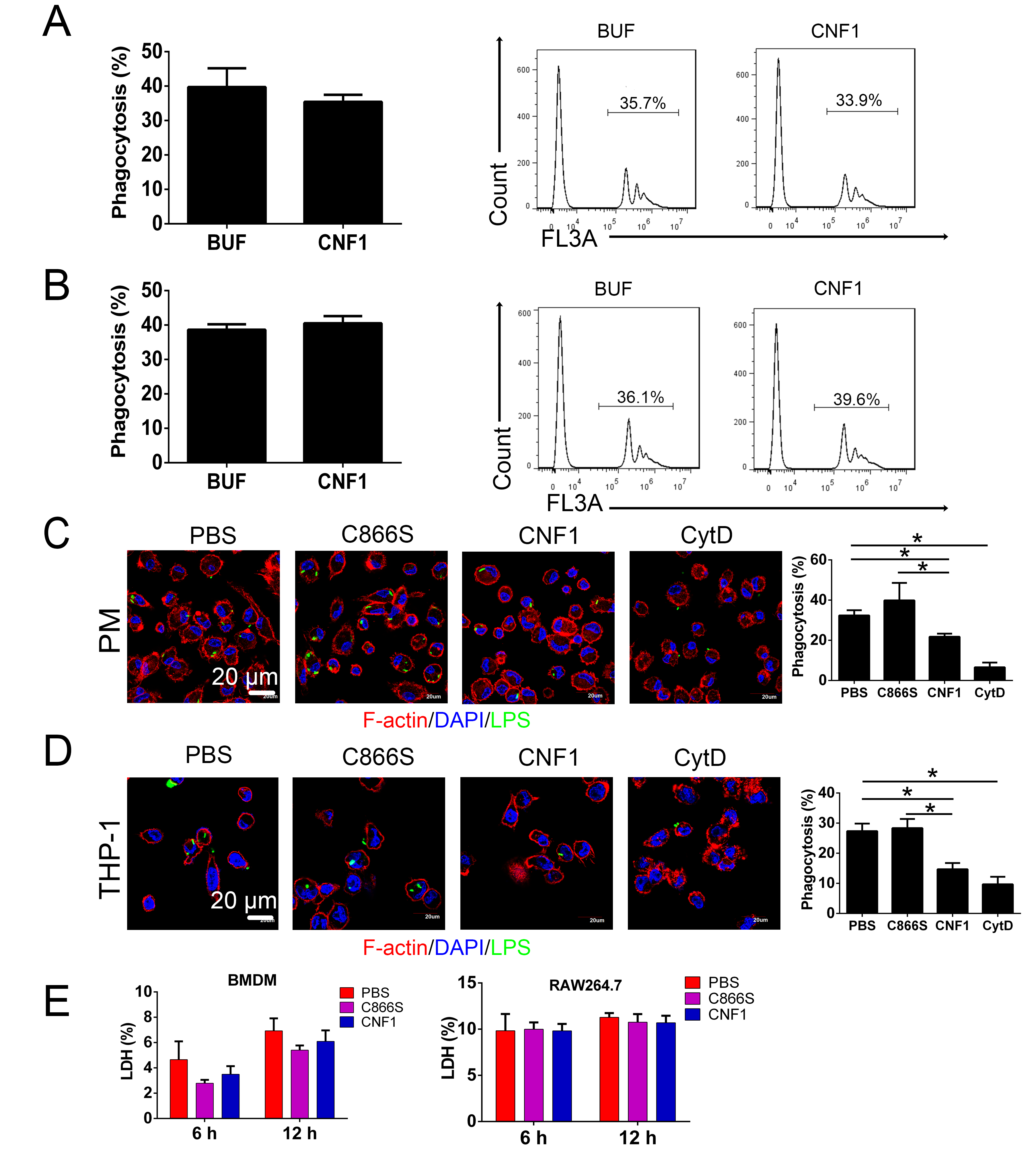

Supplement: Figure S2 — CNF1 does not reduces opsonic phagocytosis in macrophages. (A,B) FACS analysis of phagocytosis of IgG-opsonized (A) and iC3b-opsonized (B) latex beads by RAW264.7 treated with CNF1 (3 nM) and dialysis buffer for 6 h. (C,D) Immunofluorescence analysis of phagocytosis of E. coli K12 by mouse peritoneal macrophages (PM) (C) and THP-1 (D) treated with CNF1 (3 nM), PBS, C866S (3 nM, negative control), and CytD (5 μg/ml, positive control) for 6 h. Scale bar, 20 μm. Blue, nucleus; red, F-actin; Green, LPS. Bar graphs represent data from at least three independent experiments. (E) LDH assays of RAW264.7 and BMDMs treated with CNF1 (3 nM), PBS, C866S (3 nM) for 6 and 12 h. Data are from three combined independent experiments (A,B). Three hundred cells from three combined independent experiments each with two replicate wells (C,D). Data are from four combined independent experiments (E). Data are the mean ± SD, One-way ANOVA, *P < 0.05. [file Image_2.TIF]

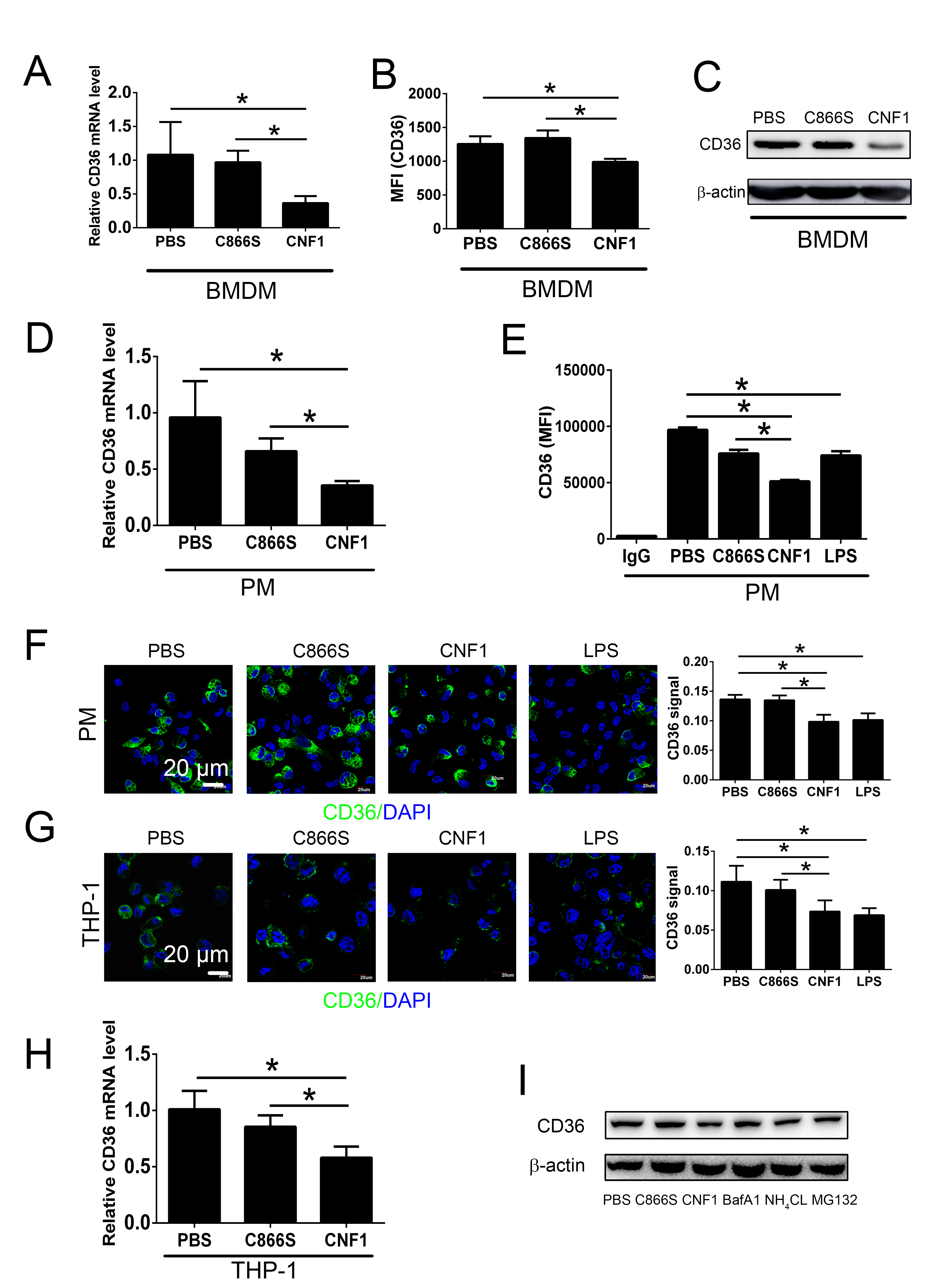

Supplement: Figure S3 — CNF1 reduces CD36 expression in PM and THP-1. (A–C) qRT-PCR (A), FACS (B), and western blotting (C) analysis of CD36 mRNA level in BMDMs treated with CNF1 (3 nM), C866S (3 nM), and PBS for 6 h. (D) qRT-PCR analysis of CD36 mRNA level in PM treated with CNF1 (3 nM), C866S (3 nM), and PBS for 6 h. (E) FACS analysis of CD36 expression in PM treated by CNF1 (3 nM), C866S (3 nM), PBS, and LPS (1 μg/ml, positive control) for 12 h. (F,G) Immunofluorescence analysis of CD36 expression in PM (D) and THP-1 (E) treated by CNF1 (3 nM), C866S (3 nM), PBS and LPS (1 μg/ml, positive control) for 6 h. Scale bar, 20 μm. Blue, nucleus; Green, CD36. (H) qRT-PCR analysis of CD36 mRNA level in THP-1 treated with CNF1 (3 nM), C866S (3 nM), and PBS for 6 h. (I) Western blotting analysis of CD36 protein level in RAW264.7 treated with CNF1 as well as lysosomal and proteasomal degradation inhibitors for 12 h (BafA1 and NH4CL for lysosomal degradation, MG132 for proteasomal degradation). Data are from three combined independent experiments each with two replicate wells (n = 6) (A,D,H). Data are from four combined independent experiments (B,E). Quantitative analysis of CD36 signal is using Image-Pro Plus, and data are from three combined independent experiments each with two fields (n = 6) (F,G). Data are the mean ± SD, One-way ANOVA, *P < 0.05. [file Image_3.TIF]

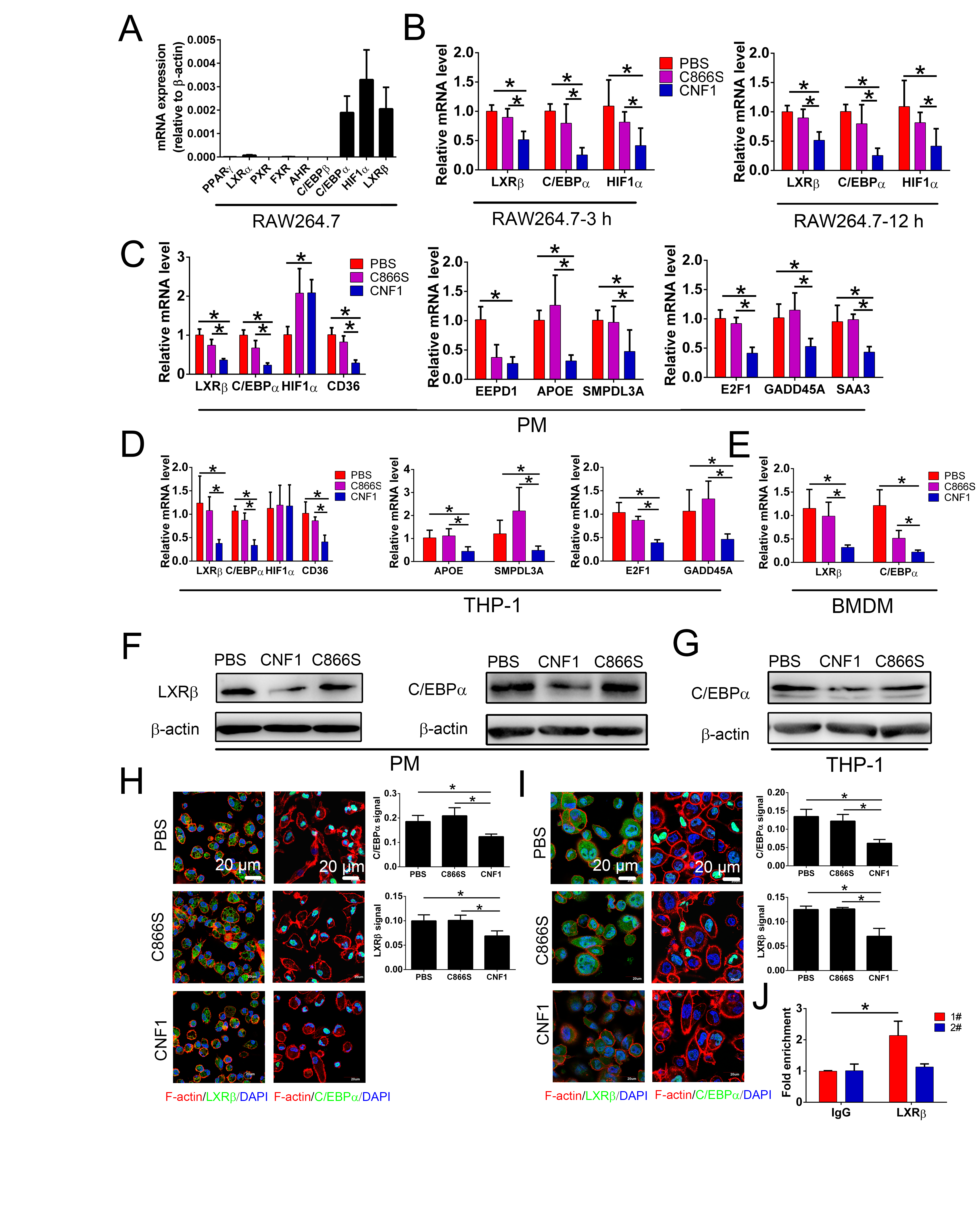

Supplement: Figure S4 — CNF1 attenuates CD36 expression by decreasing LXRβ and C/EBPα expressions in PM and THP-1. (A) qRT-PCR analysis of mRNA levels for upstream transcriptional regulators of CD36 in RAW264.7. (B) qRT-PCR analysis of mRNA levels for LXRβ, C/EBPα, HIF1α in RAW264.7 treated with CNF1 (3 nM), C866S (3 nM) and PBS for 3 and 12 h, respectively. (C,D) qRT-PCR analysis of mRNA levels for CD36, LXRβ, C/EBPα, HIF1α and genes regulated by LXRβ and C/EBPα in PM (C) and THP-1 (D) treated with CNF1 (3 nM), C866S (3 nM), and PBS for 6 h. (E) qRT-PCR analysis of mRNA levels for LXRβ and C/EBPα, HIF1α in BMDMs treated with CNF1 (3 nM), C866S (3 nM) and PBS for 6 h. (F–G) Western blotting analysis of protein levels for C/EBPα and LXRβ in PM (F) and C/EBPα in THP-1 (G) treated with CNF1 (3 nM), C866S (3 nM), and PBS for 6 h. (H–I) Immunofluorescence analysis of C/EBPα and LXRβ expressions in PM (H) and THP-1 (I) treated with CNF1 (3 nM), C866S (3 nM) and PBS for 6 h. Scale bar, 20 μm. Blue, nucleus; red, F-actin; Green, C/EBPα or LXRβ. (J) The binding of LXRβ to the two sites of the CD36 promoter in THP-1 by ChIP-qPCR analysis. Data are from three combined independent experiments each with two replicate wells (n = 6) (A–E). Quantitative analysis of C/EBPα and LXRβ signal is using Image-Pro Plus, and data are from three combined independent experiments each with two fields (n = 6) (H–I). Data are from three combined independent experiments (J). Data are the mean ± SD, One-way ANOVA, *P < 0.05. [file Image_4.TIF]

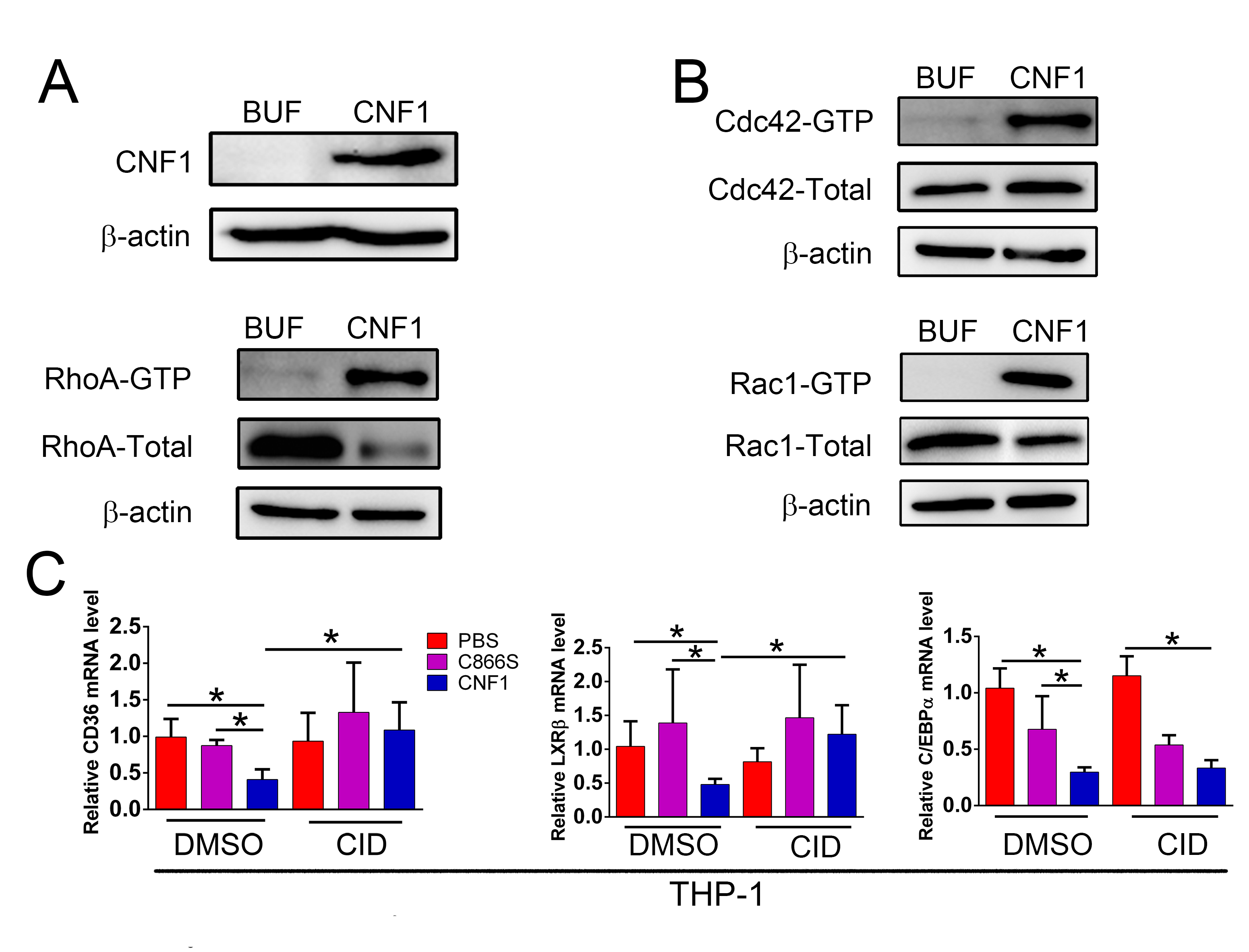

Supplement: Figure S5 — CNF1 activates Rho GTPases in macrophages and afffects LXRβ through Cdc42 in THP-1. (A) Western blotting analysis of RAW264.7 cells cultured with the CNF1 (3 nM) for 6 h. (B) Western blotting analysis of activated Rho GTPases including RhoA, Cdc42, and Rac1 after immunoprecipitation with GTP-pull down assays using specific antibodies. (C) qRT-PCR analysis of mRNA levels for CD36, LXRβ, and C/EBPα in THP-1 treated with CNF1 (3 nM), C866S (3 nM) and PBS as well as the Rac1 inhibitor EHT 1864 (EHT, 25 μM), the Cdc42 inhibitor CID44216842 (CID, 20 μM), the RhoA inhibitor CCG-1423 (CCG, 20 μM) for 12 h, respectively. Bar graphs represent data from at least three independent experiments. Data are from three combined independent experiments each with two replicate wells (n = 6) (C). Data are the mean ± SD, Two-way ANOVA, *P < 0.05. [file Image_5.TIF]
