# Supplementary material for: Cytotoxic Necrotizing Factor 1 Downregulates CD36 Transcription in Macrophages to Induce Inflammation During Acute Urinary Tract Infections
Source: Front Immunol. 2018 Aug 31;9:1987. doi: 10.3389/fimmu.2018.01987 (PMC6128224; doi:10.3389/fimmu.2018.01987)
Supplement: Table S1 — Strains and plasmids in this study. [file Table_1.docx]

**Table S1. Strains and plasmids in this study**

| **Strain or plasmid** | **Description** | **Source** |
| --- | --- | --- |
| Bacterial strain |  |  |
| CFT073  UPEC strain 11  CNF1-expressing CFT073  Vector control CFT073  UTI89  Δ*cnf1*  *E. coli* K12 | Uropathogenic *E. coli* strain  Clinical uropathogenic *E. coli* strain  CFT073 carrying pTRC99A with CNF1 (from UPEC strain 11) expression  CFT073 carrying pTRC99A  Uropathogenic *E. coli* strain  *cnf1* deletion strain derived from CFT073  *E. coli* laboratory strain | ATCC  Korczak Hospital  Our lab  Our lab  Professor Harry Mobley’s lab  Our lab  Our lab |
| *E. coli* DH5α | F^-^ *φ*80*lacZ* M15 *endA recA1* *hsdR* (r*_k_*^-^ m*_k_*^-^ ) *supE44* *thi-1* gyrA96 relA1_( *lacZYA-argF*) U169 | Beijing Dingguo Biotechmology Development Center, Beijing, China |
| *E.coli* BL21 | F- ompT hsdS B (rB- mB- ) gal  dcm (DE3) | Novagen, Merck-Millipoe |
| Plasmid |  |  |
| PET-28a(+) | T7 expression vector, KanR | Novagen, Merck-Millipoe |
| pTRC99A | Cloning vector, AmpR | Pharmacia |
| pLKO.1-puro | 3rd gene lentiviral backbone for cloing and expression of shRNA sequences, AmpR | Addgene |
| PCDH-CMV-MCS-EF1-copGFP | Mammalian Expression vector, AmpR | System Biosciences |
| pQW0001 | pET28a+ containg N-terminal 6 x histidine tagged *cnf1* from strain 11 containing C-terminal Myc | ([1](#_ENREF_1)) |
| pQW0002 | pET28a tagged C866S mutated *cnf1* from strain 11 containing C-terminal Myc | ([1](#_ENREF_1)) |
| pQW0003 | pTRC99A tagged *cnf1* from strain 11 containing C-terminal FLAG | ([1](#_ENREF_1)) |
| pQW0318 | pCDH-CMV-MCS-EF1-copGFP tagged CD36 | This work |
| pQW0319 | pLKO.1 shRNA targeting CD36 1# | This work |
| pQW0320 | pLKO.1 shRNA targeting CD36 2# | This work |

REFERENCES

1. Guo Y, Zhang Z, Wei H, Wang J, Lv J, Zhang K, et al. Cytotoxic necrotizing factor 1 promotes prostate cancer progression through activating the Cdc42-PAK1 axis. *J Pathol* (2017) 243(2):208-219. doi:10.1002/path.4940
