# Supplementary material for: Cytotoxic Necrotizing Factor 1 Downregulates CD36 Transcription in Macrophages to Induce Inflammation During Acute Urinary Tract Infections
Source: Front Immunol. 2018 Aug 31;9:1987. doi: 10.3389/fimmu.2018.01987 (PMC6128224; doi:10.3389/fimmu.2018.01987)
Supplement: Table S2 — Primers used in this study. [file Table_2.DOCX]

**Table S2. Primers used in this study**

| **Primer** | **Sequence (5’-3’)** | **Description** |
| --- | --- | --- |
| WQ0879 | CCGGAATTCATGGGCTGTGACCGG | For cloning CD36 into pCDH  -CMV-MCS-EF1-copGFP |
| WQ0880 | ATAAGAATGCGGCCGCTTATTTTATTGTTTTCGATCTGCA | For cloning CD36 into pCDH  -CMV-MCS-EF1-copGFP |
| WQ0984 | CCGGCGGATCTGAAATCGACCTTAACTCGAGTTAAGGTCGATTTCAGATCCGTTTTTG | For cloning shRNA targeting CD36 into pLKO.1 |
| WQ0985 | AATTCAAAAACGGATCTGAAATCGACCTTAACTCGAGTTAAGGTCGATTTCAGATCCG | For cloning shRNA targeting CD36 into pLKO.1 |
| WQ0986 | CCGGCCAGGGTAAGACACAGTGATACTCGAGTATCACTGTGTCTTACCCTGGTTTTTG | For shRNA targeting cloning CD36 into pLKO.1 |
| WQ0987 | AATTCAAAAACCAGGGTAAGACACAGTGATACTCGAGTATCACTGTGTCTTACCCTGG | For shRNA targeting cloning CD36 into pLKO.1 |
| WQ0759 | TTCTTCACAGCTGCCTTCTGA | For mouse CD36 qRT-PCR |
| WQ0760 | ACGTGGCCCGGTTCTAATTCA | For mouse CD36 qRT-PCR |
| WQ0856 | CAGGAGCCTGTGAGACCAAC | For mouse PPARγ qRT-PCR |
| WQ0857 | TGGCATCTCTGTGTCAACCA | For mouse PPARγ qRT-PCR |
| WQ0877 | CTGAGGACTGCAGTGTAGGAC | For human CD36 qRT-PCR |
| WQ0878 | TCACAAATCAACAGCAAGACATGA | For human CD36 qRT-PCR |
| WQ0893 | CTCAATGCCTGATGTTTCTCCT | For mouse LXRα qRT-PCR |
| WQ0894 | TCCAACCCTATCCCTAAAGCAA | For mouse LXRα qRT-PCR |
| WQ0895 | TCCAACCCTATCCCTAAAGCAA | For mouse LXRβ qRT-PCR |
| WQ0896 | GACCACGATGTAGGCAGAGC | For mouse LXRβ qRT-PCR |
| WQ0897 | GATGGAGGTCTTCAAATCTGCC | For mouse PXR qRT-PCR |
| WQ0898 | GGCCCTTCTGAAAAACCCCT | For mouse PXR qRT-PCR |
| WQ0899 | GCTTGATGTGCTACAAAAGCTG | For mouse FXR qRT-PCR |
| WQ0900 | CGTGGTGATGGTTGAATGTCC | For mouse FXR qRT-PCR |
| WQ0901 | CAAGAACAGCAACGAGTACCG | For mouse C/EBPα qRT-PCR |
| WQ0902 | GTCACTGGTCAACTCCAGCAC | For mouse C/EBPα qRT-PCR |
| WQ0903 | CGCCTTATAAACCTCCCGCT | For mouse C/EBPβ qRT-PCR |
| WQ0904 | TGGCCACTTCCATGGGTCTA | For mouse C/EBPβ qRT-PCR |
| WQ0905 | AGCCGGTGCAGAAAACAGTAA | For mouse AHR qRT-PCR |
| WQ0906 | AGGCGGTCTAACTCTGTGTTC | For mouse AHR qRT-PCR |
| WQ0907 | ACCTTCATCGGAAACTCCAAAG | For mouse HIF1α qRT-PCR |
| WQ0908 | ACTGTTAGGCTCAGGTGAAGAT | For mouse HIF1α qRT-PCR |
| WQ0966 | GGCCAGTGTTGCCTCTCTG | For mouse Ankrd37 qRT-PCR |
| WQ0967 | AGGTCAGCACCTGTTTGCAG | For mouse Ankrd37 qRT-PCR |
| WQ0968 | ATGCCCTCAGTAATGGAGAAGC | For mouse Rcor2 qRT-PCR |
| WQ0969 | CCAACTCGGATCATGCTGTCA | For mouse Rcor2 qRT-PCR |
| WQ0972 | ATGTTCCTCGCTACGTTCAAG | For mouse StAR qRT-PCR |
| WQ0973 | CCCAGTGCTCTCCAGTTGAG | For mouse StAR qRT-PCR |
| WQ0976 | GGCTGCCATCGCTCTATCC | For mouse EEPD1 qRT-PCR |
| WQ0977 | TAGCCGCTCCTGATTCACCA | For mouse EEPD1 qRT-PCR |
| WQ0978 | CTCGACTCCTCGCAGATCG | For mouse E2F1 qRT-PCR |
| WQ0979 | GATCCAGCCTCCGTTTCACC | For mouse E2F1 qRT-PCR |
| WQ0998 | CCGAAAGGATGGACACGGTG | For mouse GADD45A qRT-PCR |
| WQ0999 | TTATCGGGGTCTACGTTGAGC | For mouse GADD45A qRT-PCR |
| WQ1002 | TGCCATCATTCTTTGCATCTTGA | For mouse SAA3 qRT-PCR |
| WQ1003 | CCGTGAACTTCTGAACAGCCT | For mouse SAA3 qRT-PCR |
| WQ1004 | GGTTCTGTTTGGGTGGAGAA | For C/EBPα binding to mouse CD36 ChIP-qPCR |
| WQ1005 | TGCACATTAATCCCTTCGTG | For C/EBPα binding to mouse CD36 ChIP-qPCR |
| WQ1018 | CCAGAAAGCTAGGTCGTGGG | For human C/EBPα qRT-PCR |
| WQ1019 | ACAAACAAGGCTGAGGGTCC | For human C/EBPα qRT-PCR |
| WQ1020 | ACAGCAAGGACGACTTCCAC | For human LXRβ qRT-PCR |
| WQ1021 | TGTTGATGGCGATGAGCAGG | For human LXRβ qRT-PCR |
| WQ1022 | GGCGCATCTATGACATCACC | For human E2F1 qRT-PCR |
| WQ1023 | CCTGGGTCAACCCCTCAAG | For human E2F1 qRT-PCR |
| WQ1024 | GTTCCTTGGCGAGGCTTTTG | For human SAA1 qRT-PCR |
| WQ1025 | CTGATCACTTCTGCAGCCCA | For human SAA1 qRT-PCR |
| WQ1028 | GAGCTCCTGCTCTTGGAGAC | For human GADD45A qRT-PCR |
| WQ1029 | TCCATGTAGCGACTTTCCCG | For human GADD45A qRT-PCR |
| WQ1030 | CTGAGAAGCCCTCGAGTCAG | For human EEPD1 qRT-PCR |
| WQ1031 | CTGAGAAGCCCTCGAGTCAG | For human EEPD1 qRT-PCR |
| WQ1032 | CTCTAGAAAGAGCTGGGACCCT | For human APOE qRT-PCR |
| WQ1033 | CTTGCTCCACCTTGGCCTG | For human APOE qRT-PCR |
| WQ1034 | GTGGAGCTGCGGGACAG | For human SMPDL3A qRT-PCR |
| WQ1035 | AGTGTAAGTCAGTCACATGCCA | For human SMPDL3A qRT-PCR |
| WQ1080 | TTTCCTTCTCTTCTCCGCGT | For human HIF1α qRT-PCR |
| WQ1081 | CTGGCTGCATCTCGAGACTTT | For human HIF1α qRT-PCR |
| WQ1074 | GTTGCAATATTTGTTACTCAACTTACTG | For LXRβ binding to human CD36 ChIP-qPCR 1# |
| WQ1075 | ATGCTTTGAAAGCCTTTATCCACTTT | For LXRβ binding to human CD36 ChIP-qPCR 1# |
| WQ1078 | TAAGAAACAGAGTCTCTGGGTTTCTTA | For LXRβ binding to human CD36 ChIP-qPCR 2# |
| WQ1079 | AGACAAGCCACATAATCTCTCTACAC | For LXRβ binding to human CD36 ChIP-qPCR 2# |
